# Supply chain management with uncertainty in consumer perception of product greenness under an eco-label policy

**DOI:** 10.1038/s41598-023-40348-6

**Published:** 2023-08-21

**Authors:** Jingzhe Gao, Haixiao Wei

**Affiliations:** 1 School of Management and E-Business, Zhejiang Gongshang University, Hangzhou, 310018 Zhejiang China; 2 Modern Business Research Center of Zhejiang Gongshang University, Key Research Institute of Humanities and Social Sciences for Universities, Ministry of Education of China, Hangzhou, China; 3 School of Management, Hangzhou Dianzi University, Hangzhou, 310018 Zhejiang China

**Keywords:** Psychology and behaviour, Sustainability

## Abstract

The urgency of environmental preservation necessitates green manufacturing and supply chain advancements. This research examines a green supply chain problem influenced by eco-label policies, focusing on two prevalent market eco-label types. One allows the manufacturer to determine product greenness, while the other requires compliance with standards set by a non-governmental organization (NGO). We also explore the variability in consumer comprehension of eco-label implications and purchasing behaviors with different eco-labeled products. Through consumer utility and manufacturer profit models, we discover that the manufacturer's production and pricing choices significantly impact consumer behavior. Increased investigation costs may enhance consumer utility through improved product greenness, potentially boosting manufacturer profit via price hikes. However, if investigation costs are minimal, the NGO-label may be rejected due to decreased utility and profit. These insights could help direct supply chains by providing a theoretical foundation for green production decisions and future eco-label policies, whether determined by an NGO or the manufacturer.

## Introduction

The escalating environmental crisis, driven by swift natural resource consumption and the greenhouse effect, necessitates the adoption of green manufacturing practices^1^. Yet, companies often face a dilemma between economic growth and environmental preservation, hindering the enthusiasm for green technology development^2^. This underscores the importance of establishing apt environmental policies and regulations.

Since the 1990s, efforts from governments and NGOs have escalated to greenify industrial manufacturing and supply chains, a trend boosted by rising consumer environmental consciousness^3–7^. As per a survey, 97% of EU citizens deem environmental benefits vital, with 87% ready to partially pay for eco-friendly products^8^. It's common for environmental policies, either financial (like subsidies or taxes) or political (like caps on carbon emissions), to influence manufacturing activities^7,9–16^. Mixed regulations, like cap-and-trade policies, also exist^17–20^. This paper focuses on consumer utility and manufacturer's pricing decisions within a green supply chain under environmental regulation.

Eco-label policies are being adopted by many countries to promote green production, driven by the heightened environmental consciousness of consumers and market demand for green products^21–24^. Such policies involve third-party standard-setting for product greenness. Manufacturers can only apply eco-labels if these standards are met, assisting consumer recognition of green products. In developed countries, NGOs typically act as these third parties, termed as ‘NGO-label’ in this study. In developing nations like China, governments manage standard-setting due to their higher credibility^25^. Alternatively, manufacturers can self-report product greenness, selling green products without third-party certification, referred to as ‘self-labelling’^26,27^. This study examines the impacts of these eco-label types on the supply chain and consumer market to provide valuable managerial insights.

Consumers often struggle to perceive product greenness directly due to its multifactorial nature, including factors like emissions and recyclable content^28^. Given its subjective nature, consumer perception varies and they might distrust advertised eco-label standards. With numerous manufacturers in the market self-labeling their products, discerning true product greenness can be challenging for consumers. Determining precise greenness before purchase requires an investment of time and effort, as quick sources of information are seldom available. Consumers may only fully understand the greenness implied by the self-label after purchase and usage. NGO-labels, on the other hand, due to their uniformity and public visibility, are more easily understood. NGOs often maintain informative websites to broaden their eco-labels' impact, simplifying the investigation process. Despite these differences, consumer perception remains uncertain when encountering eco-labels, influencing purchasing behavior. Few studies have examined this behavior with eco-labeled products and its impact on manufacturers' and NGOs' decision-making, which this study addresses.

In light of the preceding discussion, this paper seeks to address the following managerial inquiries:

1. How does consumer purchasing behavior differ between these two types of green products?
2. What are the optimal pricing strategies and greenness levels for these two kinds of green products?
3. How can an NGO design the label that increases manufacturers' profitability and incentivize their adoption?

To address these questions, we establish two models, examining the managerial implications based on the results. We limit our scope to a manufacturer selling products directly to consumers, focusing on NGO, manufacturer, and consumer decisions, thus omitting retailers.

This study contributes to the understanding of eco-label policies' impact on manufacturer and consumer behavior, an area insufficiently covered in past analytical studies. It can guide manufacturers in their green production decisions and NGOs in eco-label formulation. Additionally, we captured the distinctions between mainstream eco-label types, providing a detailed comparison of the performance of self-labels and NGO-labels in a green supply chain. This sets a theoretical foundation for future eco-label policies, which is relatively uncommon in current eco-label research. Lastly, we incorporate into the analytical model the uncertain consumer understanding of the greenness of eco-labeled products and the associated cost of investigation. This factor is critical for the study and implementation of eco-labels, but is often overlooked by previous researchers. The results and propositions of the model will serve as a reference for the standardization and adoption of eco-labels.

The remainder of this paper is structured as follows. Section "Literature review" offers a literature review. Section "Problem formulation and models" establishes analytical models for the self-label and NGO-label and initiates a discussion on consumer behaviors towards different eco-labeled products. Section “Analysis and discussion” solves the models and presents several propositions for both self-label and NGO-label scenarios, followed by a comparison of these two eco-label product performances. Section “Conclusions and implications” concludes the paper, discussing managerial implications and future research directions.

## Literature review

Our work involves studying two streams of literature: environmental regulation and consumer green purchasing behavior.

### Environmental regulation

Environmental regulation is a pivotal subject within supply chain operations management. Numerous approaches, including carbon cap-and-trade, environmental taxes, green subsidies, and eco-labels, have been utilized to manage manufacturers' production activities^29–32^. Diabat and Simchi-Levi^33^ found that tighter emission caps increase supply chain costs, highlighting the need for a long-term perspective in setting carbon reduction targets. Xu et al.^34^ revealed potential inefficiencies of cap-and-trade regulation when manufacturing multiple products, given the freedom to allocate permits based on profit maximization. Cohen et al.^35^ determined that government subsidies can promote green technology adoption, but the supplier may bear significant demand risk due to the government's inability to anticipate demand uncertainty. Zhang et al.^36^ combined the remanufacturing model with government funding policies, analyzing the impact of government financial support on the decision-making of various operational entities within the closed-loop supply chain.

Given the limitations of the aforementioned environmental regulations, eco-label policies have gained increased attention^8,37–40^. Bottega and De Freitas^35^ argue that eco-label certification transforms unobservable environmental attributes into observable ones, with independent agencies like NGOs being optimal certifiers. Agatz et al.^41^ found green labels effectively guide consumer behavior, particularly amongst the eco-conscious. Houde's^2^ case study on ENERGY STAR revealed high consumer willingness to pay for certified products, despite their higher prices and efficiency.

However, alongside voluntary NGO eco-label certification, manufacturers can self-report green attributes, termed as 'self-labeling'. Murali et al.^27^ compared the impact of self-labeling and NGO-labeling on sustainable manufacturing performance, finding the credibility of manufacturers to be a key determinant of the chosen approach. They also advocated for governmental intervention in setting mandatory green standards in scenarios where manufacturers lack credibility or consumer environmental awareness is low. Auriol and Schilizzi^42^ echoed this, suggesting governments in developing countries should establish certification programs funded publicly. Lastly, Gao et al.^43^ revealed that government eco-labeling can boost supply chain profits, albeit with necessary manufacturer subsidies.

Through a review of the existing literature, it is apparent that current research on eco-labeling remains largely focused on empirical studies and case analyses, with minimal exploration of the mechanisms of eco-labeling on green production and supply chain management through mathematical models^44,45^. Furthermore, there is limited research on the impact mechanisms of different label types (NGO-label and Self-label) on consumers, enterprises, and environmental regulatory bodies. This paper constructs mathematical models for different label types and derives analytic solutions to obtain the equilibrium solutions for consumers, enterprises, and environmental regulators under various labeling scenarios. This enables a more precise analysis of the effects of eco-labeling on the decision-making of these entities, the economic and environmental benefits of the supply chain, and provides a decision-making basis for the establishment of environmental regulation and the management of green supply chains.

### Consumer green purchasing behavior

Many existing studies presume that consumers fully comprehend the greenness of a product before making purchasing decisions. However, due to the complexity of such environmental attributes, consumers might lack full understanding prior to purchase. This uncertainty could lead to post-purchase dissatisfaction, affecting consumer utility^46^. As Inman et al.^47^ noted, the discrepancy between anticipated value and actual outcome can significantly influence consumer utility.

For eco-label regulations, when manufacturers self-report product greenness using individual labels, the abundance of differing labels can lead to consumer confusion, making label comprehension more difficult^48^. Harbaugh et al.^49^ argue that such confusion might diminish the value of adopting labels altogether. Additionally, Fischer and Lyon^50^ demonstrated that competition among labels could result in minimal environmental benefits. In this context, if consumers wish to fully comprehend product greenness before purchase, substantial investigation is required. As noted by Kavaliauske et al.^51^, consumers' perception of eco-labels can be influenced by their own environmental awareness, causing the perceived value of an eco-label to vary among individuals. Taufique et al.^52^ posited that if an eco-label is designed by an independent agency like an NGO, both the NGO and manufacturers using the label could educate consumers, thereby encouraging environmentally friendly behavior. As previously discussed, NGO-set eco-labels provide consumers with more accessible ways to understand product greenness, such as through NGO websites, making it simple and cost-effective for consumers to become well-informed.

In summary, few studies have examined the distinct consumer behaviors towards manufacturer self-labels and NGO eco-labels, and the resulting effects on sustainable manufacturing under differing label regulations. While some literature explores consumer purchasing behavior for eco-labeled products, there is a lack of mathematical models illustrating the utility of consumer purchasing behaviors under manufacturer self-labels and NGO eco-labels^5,53^. Table 1 summarizes the above previous works and compares them to the present study. Importantly, when consumers need to investigate a product prior to purchasing, this process incurs an investigation cost not considered when making a direct purchase. This paper aims to fill this research gap by exploring the optimal decisions manufacturers make under these two types of label regulations. By comparing these different label types, this study also seeks to guide NGOs in implementing eco-label regulations to encourage more manufacturers to adopt eco-labels.

**Table 1:** Comparisons between this study and related literature.

Papers	Decision maker			Environmental regulation				Research method		
	Enterprise	Consumer	NGO/Governmant	Tax\Subsidy	Carbon cap	Eco-label		Empirical research	Case study	Mathematical model
						Self-label	NGO-label			
Cohen et al. ^35^	✓	✓		✓						✓
Heyes and Martin ^38^	✓		✓				✓	✓		
Gao et al. ^43^	✓			✓			✓		✓	
Taufique et al. ^52^		✓					✓		✓	
Chai et al. ^17^	✓		✓		✓			✓		
Nadar and Ertürk ^21^	✓						✓		✓	
Agatz et al. ^41^	✓			✓			✓	✓		
Roh et al. ^1^	✓			✓	✓			✓		
Houde ^2^	✓						✓	✓		
Barkemeyer et al. ^5^	✓						✓		✓	
Liu et al. ^19^	✓		✓		✓			✓		
Zhu et al. ^22^	✓		✓				✓		✓	
This study	✓	✓	✓			✓	✓			✓

According to Table 1, from the perspective of the research subject, past studies on green supply chain management have primarily focused on decision-making within the supply chain entities, neglecting the behaviors of indirect participants in supply chain emission reduction (consumers, governments, NGOs). There has been inadequate consideration of the impacts at the level of consumer environmental awareness and green consumption paths, which are no longer suitable for today's complex emission reduction situation and supply chain systems. From the content perspective, existing literature tends to concentrate on studying conventional environmental regulations such as environmental taxes, green subsidies, carbon quotas, or third-party label certification. The influence of different types of eco-labels, which are popular today, on the decision-making mechanisms of both direct and indirect supply chain entities, has received little attention. From a methodological perspective, most research on environmental regulation adopts empirical studies or case analyses. There is a noticeable scarcity of research using the construction of mathematical models to dissect the driving mechanisms of environmental regulation on production and pricing decisions within supply chain enterprises.

Addressing current gaps in the field, this paper's key innovations include:

1. For top-level eco-label regulation design, we establish and compare models for self-label and NGO-label systems to provide a decision-making framework that balances economic and environmental goals.
2. For green supply chain decision-making, we consider the varying degrees of economic and environmental concerns across supply chain entities. This approach informs production and pricing decisions, maximizing eco-labels' potential in fostering supply chain sustainability amidst complex structures and environmental scenarios.
3. For driving green consumption, we address the complexity of green product information and consumer environmental awareness uncertainty. Through eco-label market role modeling and analysis, we not only ensure the sustainable development of the green consumer market but also stimulate supply chain greening from the end-market perspective.

## Problem formulation and models

In this section, we mathematically describe our problem and establish our models. The notations used in this study are presented in Table 2. Owing to the surge in consumer environmental consciousness, product greenness can impact consumer utility, thus motivating manufacturers to produce eco-friendly products. In our study, a single manufacturer supplies a specific product directly to consumers. Analogous to Syam et al.^54^, we use a parameter $q\in \left[{0,1}\right]$ to denote product greenness.

**Table 2:** Symbols and notations.

$q$	Product greenness ($0\le q\le 1$)
$x$	The gap between the consumers’ perception of product greenness and that reported by the manufacturer’s self-label ($0\le x\le 1$)
$v$	Consumer valuation of the product ($v\ge 0$)
$b$	Consumer preference coefficient for product greenness ($b\ge 0$)
$p$	Product price ($p\ge 0$)
$c$	Investigation cost for the self-labeled product ($c\ge 0$)
$D$	Consumer demand ($D\ge 0$)
$\alpha$	Green cost coefficient ($\alpha \ge 0$)
$U$	Consumer utility ($U\ge 0$)
$\pi$	Manufacturer profit ($\pi \ge 0$)

**Figure 1:**
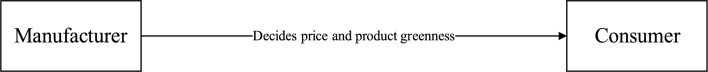
Self-label structure.

**Figure 2:**
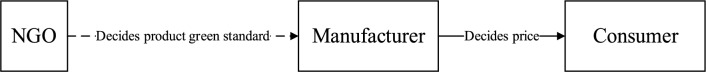
NGO-label structure.

For manufacturers, there are two strategies to communicate product greenness to consumers. One is to self-declare the greenness on the product’s packaging using a green label, a method we refer to as 'self-labeling' following Murali et al.^27^. Alternatively, they can seek external certifications from environmental NGOs. In this study, we consider a single NGO. The NGO sets a benchmark level of greenness based on market research. Only products meeting this standard can receive certification and bear the NGO’s green label on their packaging. This strategy is termed 'NGO-labeling'. Figures 1 & 2 illustrate the supply chain structures for these two strategies.

### Self-label scenario

As shown in Fig. 1, when the manufacturer opts for self-labeling, it has the autonomy to establish the product's greenness level and price. In this paper, we have made the following assumption:

#### Assumption 1

According to the studies of Brécard^48^ and Harbaugh et al.^49^, we assume that consumers' perceptions of the greenness of a product are uncertain.

This means when the manufacturer or NGO reports a greenness level, $q$, consumers may not fully believe this is the true level. They formulate their own understanding, generally perceiving the greenness level to be lower than what the manufacturer claims. Therefore, it's common for manufacturers to exaggerate the greenness level on the self-label to make their product more appealing. To mathematically represent this situation, we introduce a random variable, $x$, that indicates the gap between consumers' perception of product greenness and the greenness level reported on the manufacturer's self-label. The perceived greenness can be calculated as $q-x$. Due to the intricacies involved in the calculation of greenness, consumers generally can't precisely determine the level of product greenness before making a purchase, leading to a degree of uncertainty in their perceptions. We model this uncertainty using a probability distribution. Following the approach of Syam et al.^27^, we use a uniform distribution to describe the variable $x$.

In this self-labeling scenario, the diversity of labels across different manufacturers can confuse consumers, making it difficult for them to understand the product's greenness. If consumers want to ascertain the precise value of $x$(i.e., fully understand the product's greenness) before purchasing the product, they incur an investigation cost, $c$. Here, we introduce another assumption.

#### Assumption 2

Similar to the paper of Kuksov and Villas-Boas^55^, we assume that the consumer's utility function is a linear function concerning the product's price and the consumer's perception of the product's green attributes.

When consumers opt to investigate before purchasing the product, their utility is influenced by the actual perceived greenness, $q-x$. This utility can be formulated as $U=v+b(q-x)-p-c$, where $v$ represents the consumers' valuation of the product, $b$ is the consumers' preference coefficient for the level of greenness, and $p$ is the product's price. Consistent with the study by Zhu and He^56^, we can normalize consumer sensitivity to price as 1. The use of a linear function to represent consumer utility is common in the literature^47,54,57^. However, consumers may experience regret after deciding to investigate the product. If, after investigation, they find the product unsatisfactory and choose not to purchase, they still incur the investigation cost, resulting in a utility of $U=-c$. Alternatively, consumers can choose not to investigate the product and purchase it directly. In this case, they will only learn about their perception of the product's greenness after using it. If they buy the product without investigation, we let $x$ vary over the interval $\left[{0,1}\right]$. A situation where $q-x<0$ suggests that the consumer perceives the product as potentially harmful to the environment after investigating. Therefore, the expected utility in the case of direct purchase without investigation can be calculated as ${\int }_{0}^{1}v+b(q-x)-p dx=v+bq-p-\frac{1}{2}b$.

In this scenario, the manufacturer seeks to maximize its profit by determining the optimal price, $p$, and level of greenness, $q$. Drawing from the work by Ghosh and Shah^58^, we can express the manufacturer's objective function as $Max \pi =pD-\alpha {q}^{2}$. Here, $\alpha {q}^{2}$ is the cost of green production^16^, and $\alpha$ represents the level of green production technology. If the technology is advanced, producing green products would be cheaper, and vice versa. Given that the range of $q$ is $\left[{0,1}\right]$, the upper bound of the green production cost is $\alpha$, which should be significantly large considering the complexity involved in producing a fully green product. Thus, we have made a third assumption.

#### Assumption 3

We assume that $\alpha >v+b$, which simplifies our calculations without altering the managerial implications^43^.

It's important to note that the cost related to greenness, represented as a quadratic term in this study, is independent of demand. This is due to the nature of green technology^58^ and is a common assumption in the literature^9,59^.

That's a clear way of notating the different variables in this scenario. Using these subscript notations will certainly make the remainder of the modelling process more understandable. To reiterate: The subscripts $S$ and $N$ are used to indicate the self-label and NGO-label scenarios, respectively. The subscripts $i$ and $d$ are used to denote consumers who investigate and do not investigate the product, respectively. The subscripts $b$ and $o$ refer to buying and no-buying scenarios. For example, ${U}_{Sdb}$ represents the utility of consumers who do not investigate the product but buy it directly in the self-label scenario. On the other hand, ${\pi }_{No}$ represents the profit when consumers do not buy the product in the NGO-label scenario. These notations will streamline the discussion of these models and the implications of the analysis.

Upon evaluating a green product, consumers simply need to contrast the two utilities in question, thereby informing their purchase decision.

$$ \left\{ \begin{gathered}   U_{{Sib}}  = v + b(q - x) - p - c{\text{ if the consumers buy the product}} \hfill \\   U_{{Sio}}  =  - c{\text{ if the consumers do not buy the product}} \hfill \\  \end{gathered}  \right. $$

For a consumer, the decision to purchase hinges on whether ${U}_{Sib}$ exceeds ${U}_{Sio}$. If it does, they choose to buy the product post-evaluation. Conversely, if it doesn't, they opt not to. Furthermore, having assessed the product, their utility is now at $-c$. Given perceptual uncertainties, the anticipated utility of investigation, ${U}_{Si}$, is expressed as an integral of ${U}_{Sib}$ and ${U}_{Sio}$ with respect to $x$.

Should consumers opt against investigating the green product, they must then compare these two utilities to make an informed decision.

$$ \left\{ \begin{gathered}   U_{{Sdb}}  = v + bq - p - \frac{1}{2}{\text{b  if the consumers buy the product}} \hfill \\   U_{{Sdo}}  = 0{\text{ if the consumers do not buy the product}} \hfill \\  \end{gathered}  \right. $$

Mirroring the previous case, consumers will purchase the product if ${U}_{Sdb}\ge {U}_{Sdo}$, and otherwise will not. However, when consumers forgo product investigation before making the buying decision, their expected utility remains unaffected by $x$, thus, the demand is binary—either 1 or 0. This implies a scenario where either all consumers will buy the product or none will. Consequently, we deduce that ${U}_{Sd}$ equates to either ${U}_{Sdb}$ or ${U}_{Sdo}$.

In the self-labeling scenario, the consumer's decision process bifurcates into two stages: the initial stage of deciding whether to investigate the product and the subsequent stage of deciding whether to make a purchase. In the first stage, consumers must weigh the expected utilities of investigating versus not investigating the product, comparing ${U}_{Si}$ with ${U}_{Sd}$. The decision to investigate the product hinges on meeting both ${U}_{Si}>{U}_{Sdb}$ and ${U}_{Si}\ge {U}_{Sdo}$ conditions. For the second stage, if consumers investigate the product and decide to buy, ${U}_{Sib}\ge {U}_{Sio}$ must hold true. Alternatively, if consumers bypass investigation yet decide to purchase, conditions ${U}_{Si}\le {U}_{Sdb}$ and ${U}_{Sdb}\ge {U}_{Sdo}$ must be met. Consequently, we use the following proposition to explicate the conditions guiding consumer purchasing decisions.

#### Proposition 1

(1) Suppose that $c\le \frac{b}{8}$; when $0\le p<v+bq-b+\sqrt{2bc}$, all consumers opt for purchasing without investigating the product. However, if $v+bq-b+\sqrt{2bc}\le p\le v+bq-\sqrt{2bc}$, consumers would investigate the product, and those whose $x$ is in the range of $0\le x\le \frac{v-p}{b}+q$ would purchase it, while others would abstain. (2) Suppose that $c>\frac{b}{8}$; when $0\le p\le v+bq-\frac{1}{2}b$, all consumers proceed with buying without any prior investigation. In other situations, they refrain from purchasing.

Applying the aforementioned conditions, we derive the corresponding utilities and demands for each situation. With these $p$ and $q$ constraints, we can then compute the manufacturer's optimal decisions. The proof of this proposition and the optimal decisions are presented in the Appendix. Proposition 1 reveals that consumer product investigation only occurs when the associated cost is minimal. If the cost exceeds $\frac{b}{8}$, consumers either elect to purchase the product without prior investigation or refrain from buying altogether. Furthermore, this proposition indicates that consumers' purchasing behavior towards self-label products is determined by both the investigation cost and product price. Therefore, to some extent, the manufacturer can influence consumers' purchasing behavior by implementing promotional marketing strategies to lower the investigation cost and making strategic pricing decisions.

Up to this point, we have constructed the model for the self-label scenario. In the following section, we will explore the model within the context of an NGO-label scenario.

### NGO-label scenario

In this scenario, while the product is manufactured by the producer, its greenness level is ascertained by an NGO. The manufacturer can only set the price, and must conform to the NGO's greenness level to obtain certification. We do not consider the possibility of a manufacturer exceeding the NGO's standard. In practice, an NGO-label is a uniform identifier for a product class, and any attempts to surpass the standard still result in the same label, leading to unnecessary additional costs without attracting more consumers. Thus, typically, the manufacturer adheres strictly to the green standard^20^. A unified green label from an NGO brings numerous benefits: it is perceived as more authoritative given the non-profit nature of the NGO, and the uniformity of the label simplifies consumer understanding of the product's greenness. Thus, we have the following assumption.

#### Assumption 4

We assume a zero investigation cost in this scenario, signifying consumers always have precise knowledge of $x$ before purchasing. They are therefore always aware of their perception value, which is $q-x$ for product greenness.

If consumers purchase the product, the utility is $U=v+b(q-x)-p$, eradicating any regret. The consumer decision process is now singular, consisting only of the purchase decision. Consequently, we compare the following two utilities.

$$\left\{\begin{array}{l}{U}_{Nb}=v+b(q-x)-p\text{ if the consumers buy the product}\\ {U}_{No}=0\text{ if the consumers do not buy the product}\end{array}\right.$$

If consumers elect to purchase the product, the condition ${U}_{Nb}\ge {U}_{No}$ must be met. Failing this, they would refrain from buying. This leads us to the following proposition.

#### Proposition 2

(1) If $p<v+bq-b$, all consumers opt to buy the product. (2) If $v+bq-b\le p\le v+bq$, the consumers whose $x$ falls within the range of $0\le x\le \frac{v-p}{b}+q$ would proceed with the purchase. In other cases, they would abstain. (3) If $p>v+bq$, no consumers would forgo the product altogether.

Using the outlined conditions, we can derive the respective utilities and demands for each situation. Subsequently, the manufacturer's optimal decisions can be calculated considering these constraints for $p$. The proof of this proposition and optimal decisions are found in the Appendix. Proposition 2 suggests that only when the price is exceptionally low, will all consumers buy the product irrespective of their $x$ value. As the price escalates, consumer behavior becomes contingent upon the value of $x$. However, if the price surpasses $v+bq$, consumer interest is entirely lost. Consequently, in scenarios similar to those involving self-label products, the manufacturer can likewise influence the purchasing behavior of consumer groups towards NGO-label products through strategic product pricing decisions.

We have thus completed the modeling of both scenarios. In the subsequent section, we will dissect the results, draw comparisons, and derive a series of propositions and intriguing insights.

## Analysis and discussion

### Self-label scenario

So far, we have determined all solutions in every situation, irrespective of the consumers' inclination to investigate the product. These decisions regarding price and greenness are merely optional solutions in the self-label scenario, implying that in reality, the manufacturer must compare the profits across these solutions and choose the one that maximizes profit. By setting the price and level of greenness, the manufacturer can influence consumer investigation behaviors. Consequently, in the self-label scenario, there must ultimately be a single optimal solution. Further details are outlined as follows.

#### Proposition 3

When $c\le {\min}(\frac{((-4b+2v)\alpha +{b}^{2}{)}^{4}}{32{\alpha }^{2}{b}^{3}(-4\alpha +b{)}^{2}},\frac{b}{8})$, the manufacturer would make the decision $$\left\{\begin{array}{l}{p}_{Si}^{*}=\frac{2\alpha v}{4\alpha -b}\\ {q}_{Si}^{*}=\frac{v}{4\alpha -b}\end{array}\right.$$, with corresponding profit ${\pi }_{Si}^{*}=\frac{\alpha {v}^{2}}{(4\alpha -b)b}$ and utility ${U}_{Si}^{*}=\frac{2{\alpha }^{2}{v}^{2}}{(b-4\alpha {)}^{2}b}-c$. When $\frac{((-4b+2v)\alpha +{b}^{2}{)}^{4}}{32{\alpha }^{2}{b}^{3}(-4\alpha +b{)}^{2}}<c\le \frac{b}{8}$, the manufacturer would make the decision $$\left\{\begin{array}{l}{p}_{Sd}^{*}=\frac{2\sqrt{2bc}\alpha -2b\alpha +2v\alpha +{b}^{2}}{2\alpha }\\ {q}_{Sd}^{*}=\frac{b}{2\alpha }\end{array}\right.$$, with corresponding profit ${\pi }_{Sd}^{*}=\frac{4\sqrt{2bc}\alpha +4(v-b)\alpha +{b}^{2}}{4\alpha }$ and utility ${U}_{Sd}^{*}=\frac{b}{2}-\sqrt{2bc}$. When $c>\frac{b}{8}$, the manufacturer would make the decision $$\left\{\begin{array}{l}{p}_{Sd}^{*}=-\frac{b\alpha -2v\alpha -{b}^{2}}{2\alpha }\\ {q}_{Sd}^{*}=\frac{b}{2\alpha }\end{array}\right.$$, with corresponding profit ${\pi }_{Sd}^{*}=\frac{(4v-2b)\alpha +{b}^{2}}{4\alpha }$ and utility ${U}_{Sd}^{*}=0$.

The proof can be found in the Appendix. We observe that the manufacturer can choose between two or three decisions. Although, as shown in Proposition 1, the manufacturer appears to influence consumers' purchasing behavior and whether they investigate the product prior to purchase through production pricing decisions, in reality, the greenness level of the manufacturer's products and pricing decisions are determined by the consumers' investigation costs. Therefore, the trajectory of consumers' purchasing behavior towards self-label products ultimately depends on the scale of their product investigation costs. If $\frac{((-4b+2v)\alpha +{b}^{2}{)}^{4}}{32{\alpha }^{2}{b}^{3}(-4\alpha +b{)}^{2}}\le \frac{b}{8}$, the manufacturer has three decision points as the investigation cost increases from zero to infinity. When $c\le {\min}(\frac{((-4b+2v)\alpha +{b}^{2}{)}^{4}}{32{\alpha }^{2}{b}^{3}(-4\alpha +b{)}^{2}},\frac{b}{8})$, the manufacturer's decision will prompt consumers to investigate the product and buy it. The product price and greenness are not influenced by the investigation cost in this case. As the cost rises and meets $\frac{((-4b+2v)\alpha +{b}^{2}{)}^{4}}{32{\alpha }^{2}{b}^{3}(-4\alpha +b{)}^{2}}<c\le \frac{b}{8}$, the manufacturer alters the decision, steering consumers to directly buy the product without investigation. At this point, greenness remains constant, but the price is a growing function of $c$. When the cost escalates to satisfy $c>\frac{b}{8}$, the manufacturer again changes the decision, making consumers buy the product directly. The final price and greenness are unaffected by the cost. However, if $\frac{((-4b+2v)\alpha +{b}^{2}{)}^{4}}{32{\alpha }^{2}{b}^{3}(-4\alpha +b{)}^{2}}>\frac{b}{8}$, the interval $\frac{((-4b+2v)\alpha +{b}^{2}{)}^{4}}{32{\alpha }^{2}{b}^{3}(-4\alpha +b{)}^{2}}<c\le \frac{b}{8}$ cannot exist. Consequently, as the cost increases, there are two decision points for the manufacturer. The decision switch would occur at $c=\frac{b}{8}$. This hinges on the magnitude of $\frac{((-4b+2v)\alpha +{b}^{2}{)}^{4}}{32{\alpha }^{2}{b}^{3}(-4\alpha +b{)}^{2}}$ and $\frac{b}{8}$. They are contingent upon the values of $v$, $b$ and $\alpha$. The first two parameters depend on consumers, and the last parameter depends on the level of green production technology. Thus, the manufacturer's decisions and whether consumers would investigate the product are determined by the market conditions and green technology levels.

There are fascinating insights derived from these results that highlight certain characteristics of the manufacturer's actions within the self-label scenario.

#### Proposition 4

(1) If $\frac{(4\alpha -b-\sqrt{2\alpha (4\alpha -b)})b}{2\alpha }\le v<\frac{(4\alpha -b)b}{2\alpha }$, the expected utility first declines with $c$ in the range of $\left[0,\frac{((-4b+2v)\alpha +{b}^{2}{)}^{4}}{32{\alpha }^{2}{b}^{3}(-4\alpha +b{)}^{2}}\right]$, then rises at the point $c=\frac{((-4b+2v)\alpha +{b}^{2}{)}^{4}}{32{\alpha }^{2}{b}^{3}(-4\alpha +b{)}^{2}}$, and subsequently decreases in the interval $c$ in $(\frac{((-4b+2v)\alpha +{b}^{2}{)}^{4}}{32{\alpha }^{2}{b}^{3}(-4\alpha +b{)}^{2}},\frac{b}{8}]$. Eventually, the utility falls to zero and stays there for $c$ in $\left(\frac{b}{8},+\infty \right)$ (2) If $v<\frac{(4\alpha -b-\sqrt{2\alpha (4\alpha -b)})b}{2\alpha }$ or $v\ge \frac{(4\alpha -b)b}{2\alpha }$, the expected utility consistently decreases for $c$ in $\left[0,\frac{b}{8}\right]$, and eventually settles at zero for $c$ in $\left(\frac{b}{8},+\infty \right)$.

The demonstration of the proof is presented in the Appendix. Our proposition reveals that consumer's expected utility largely diminishes as $c$ increases. However, there still exist some intriguing anomalies. The utility surge at point $c=\frac{((-4b+2v)\alpha +{b}^{2}{)}^{4}}{32{\alpha }^{2}{b}^{3}(-4\alpha +b{)}^{2}}$ can be attributed to Proposition 3. Here, if the term is $\frac{((-4b+2v)\alpha +{b}^{2}{)}^{4}}{32{\alpha }^{2}{b}^{3}(-4\alpha +b{)}^{2}}\le \frac{b}{8}$, the manufacturer decides $$\left\{\begin{array}{c}{p}_{Si}^{*}=\frac{2\alpha v}{4\alpha -b}\\ {q}_{Si}^{*}=\frac{v}{4\alpha -b}\end{array}\right.$$ for $c$ in $\left[0,\frac{((-4b+2v)\alpha +{b}^{2}{)}^{4}}{32{\alpha }^{2}{b}^{3}(-4\alpha +b{)}^{2}}\right]$. The decision becomes $$\left\{\begin{array}{l}{p}_{Sd}^{*}=\frac{2\sqrt{2bc}\alpha -2b\alpha +2v\alpha +{b}^{2}}{2\alpha }\\ {q}_{Sd}^{*}=\frac{b}{2\alpha }\end{array}\right.$$ for $c$ in $(\frac{((-4b+2v)\alpha +{b}^{2}{)}^{4}}{32{\alpha }^{2}{b}^{3}(-4\alpha +b{)}^{2}},\frac{b}{8}]$. When $\frac{(4\alpha -b-\sqrt{2\alpha (4\alpha -b)})b}{2\alpha }\le v<\frac{(4\alpha -b)b}{2\alpha }$, the term is $\frac{((-4b+2v)\alpha +{b}^{2}{)}^{4}}{32{\alpha }^{2}{b}^{3}(-4\alpha +b{)}^{2}}\le \frac{b}{8}$, and ${q}_{Si}^{*}=\frac{v}{4\alpha -b}<{q}_{Sd}^{*}=\frac{b}{2\alpha }$, signifying an increase in product greenness. This triggers an upswing in utility to a higher level. The manufacturer controls consumer choice. For $c>\frac{b}{8}$, as consumers cannot assess the product and must purchase it outright, the manufacturer simply ensures the utility is non-negative. Hence, the equilibrium occurs at ${U}_{Sd}^{*}=0$. In addition, Proposition 4 also suggests that the most important utility that consumers care about when purchasing self-label products will be significantly influenced by changes in the investigation costs. Hence, any marketing strategies from the manufacturer aimed at reducing these costs can be extremely effective for product sales.

**Figure 3:**
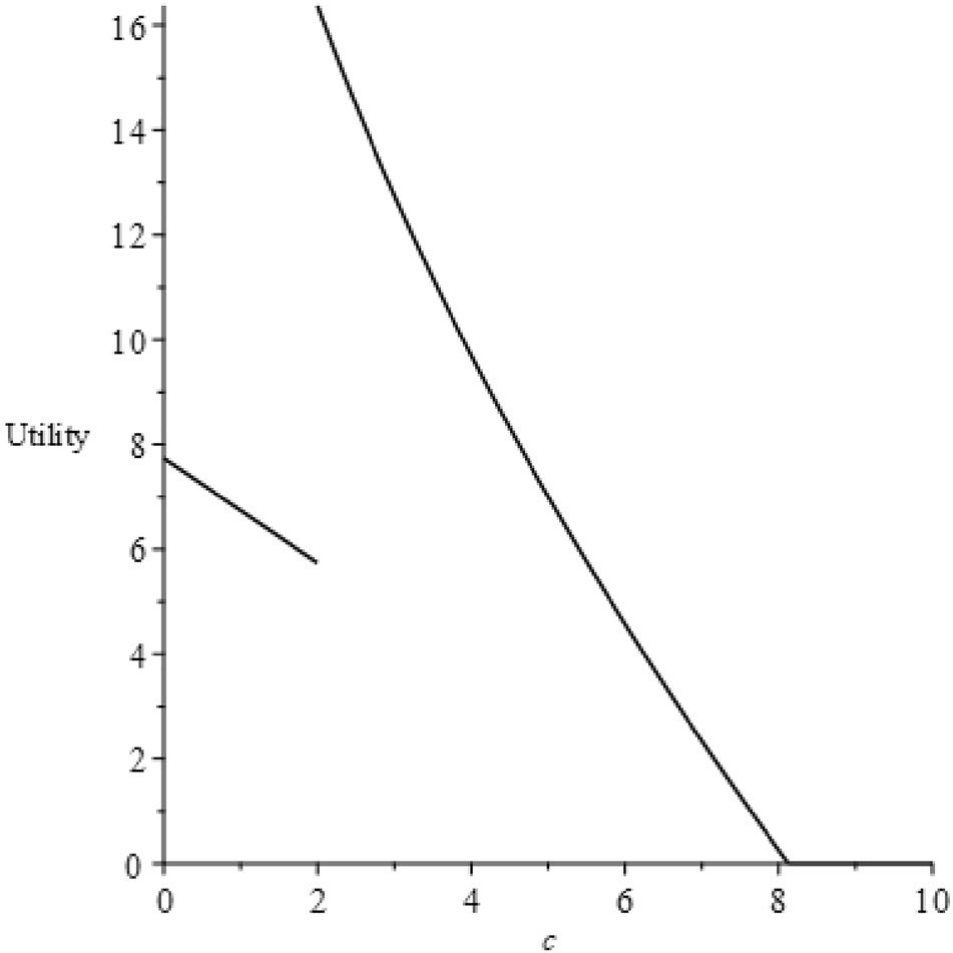
Utility changes according to $c$.

The initial segment of this proposition is graphically elucidated in our figures. Given that the model's parameters can be abstract and challenging to derive from real-world industries, we make assumptions: $v=60$, $b=65$ and $\alpha =300$. With these values, we find that the condition $\frac{(4\alpha -b-\sqrt{2\alpha (4\alpha -b)})b}{2\alpha }\le v<\frac{(4\alpha -b)b}{2\alpha }$ holds true. Figure 3 depicts the trends of utility in relation to $c$.

The utility, as shown, initially declines and then ascends to a peak at point $c=\frac{((-4b+2v)\alpha +{b}^{2}{)}^{4}}{32{\alpha }^{2}{b}^{3}(-4\alpha +b{)}^{2}}=1.683$, after which it dwindles until $c=\frac{b}{8}$, ultimately settling at zero. This trend can be attributed to a change in the manufacturer's optimal decision at $c=1.683$, altering the functional form of utility. Despite this, both utility forms remain decreasing functions of $c$, thus it can only rise at this point. For $c>\frac{b}{8}$, the manufacturer would adjust its decision again, reducing consumers to buying the product without prior scrutiny, thus maintaining the utility consistently at zero.

#### Proposition 5

(1) If $v<\frac{(4\alpha -b-\sqrt{2\alpha (4\alpha -b)})b}{2\alpha }$, product greenness remains constant for $c$ in $\left[0,\frac{b}{8}\right]$. It then surges to a higher level, persisting within $c$ in $\left(\frac{b}{8},+\infty \right)$. (2) If $\frac{(4\alpha -b-\sqrt{2\alpha (4\alpha -b)})b}{2\alpha }\le v<\frac{(4\alpha -b)b}{2\alpha }$, product greenness is steady for $c$ in $\left[0,\frac{((-4b+2v)\alpha +{b}^{2}{)}^{4}}{32{\alpha }^{2}{b}^{3}(-4\alpha +b{)}^{2}}\right]$. Then, it jumps to a high level, staying there within $c$ in $\left(\frac{((-4b+2v)\alpha +{b}^{2}{)}^{4}}{32{\alpha }^{2}{b}^{3}(-4\alpha +b{)}^{2}},+\infty \right)$. (3) If $\frac{(4\alpha -b)b}{2\alpha }<v\le \frac{(4\alpha -b+\sqrt{2\alpha (4\alpha -b)})b}{2\alpha }$, product greenness remains static for $c$ in $\left[0,\frac{((-4b+2v)\alpha +{b}^{2}{)}^{4}}{32{\alpha }^{2}{b}^{3}(-4\alpha +b{)}^{2}}\right]$. Afterwards, it declines to a lower level, persisting there within $c$ in $\left(\frac{((-4b+2v)\alpha +{b}^{2}{)}^{4}}{32{\alpha }^{2}{b}^{3}(-4\alpha +b{)}^{2}},+\infty \right)$. (4) If $v>\frac{(4\alpha -b+\sqrt{2\alpha (4\alpha -b)})b}{2\alpha }$, product greenness is constant for $c$ in $\left[0,\frac{b}{8}\right]$. It then drops to a lower level, remaining there within $c$ in $\left(\frac{b}{8},+\infty \right)$.

**Figure 4:**
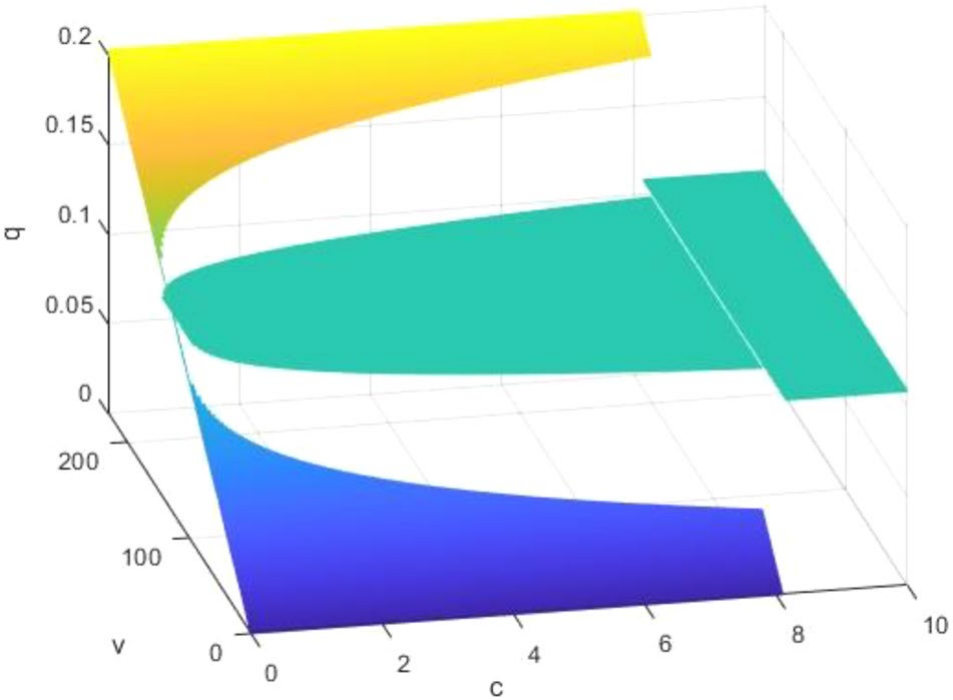
How greenness changes according to $v$ and $c$.

The proof is provided in the Appendix. As per Proposition 3, we observe that the optimal level of greenness remains largely unimpacted by $c$. Only a change in the manufacturer's decision could influence greenness. Therefore, although compared to NGO-label products, consumers face decisions regarding investigation costs and whether to investigate before purchasing when buying self-label products, in reality, this does not affect the manufacturer's decisions about the greenness of the product. Consequently, changes would occur only at the aforementioned $c$ points. Assuming $b=65$ and $\alpha =300$, Fig. 4 visualizes the variations in greenness relative to $v$ and $c$.

In this context, whether a higher investigation cost promotes a greener product is dictated by the relationship between $v$ and $\frac{(4\alpha -b)b}{2\alpha }$. This relationship could be interpreted as the interplay between market conditions and green technology advancements.

#### Proposition 6

(1) If $\frac{(4\alpha -b-\sqrt{2\alpha (4\alpha -b)})b}{2\alpha }\le v\le \frac{(4\alpha -b+\sqrt{2\alpha (4\alpha -b)})b}{2\alpha }$, profit remains steady for $c$ in $\left[0,\frac{((-4b+2v)\alpha +{b}^{2}{)}^{4}}{32{\alpha }^{2}{b}^{3}(-4\alpha +b{)}^{2}}\right]$, then increases within $c$ in $(\frac{((-4b+2v)\alpha +{b}^{2}{)}^{4}}{32{\alpha }^{2}{b}^{3}(-4\alpha +b{)}^{2}},\frac{b}{8}]$. Subsequently, it again becomes constant for $c$ in $\left(\frac{b}{8},+\infty \right)$. The overall trend is continuous. (2) If $v<\frac{(4\alpha -b-\sqrt{2\alpha (4\alpha -b)})b}{2\alpha }$ or $v>\frac{(4\alpha -b+\sqrt{2\alpha (4\alpha -b)})b}{2\alpha }$, profit is constant for $c$ in $\left[0,\frac{b}{8}\right]$, after which it falls to a lower level and stays there within $c$ in $\left(\frac{b}{8},+\infty \right)$.

**Figure 5:**
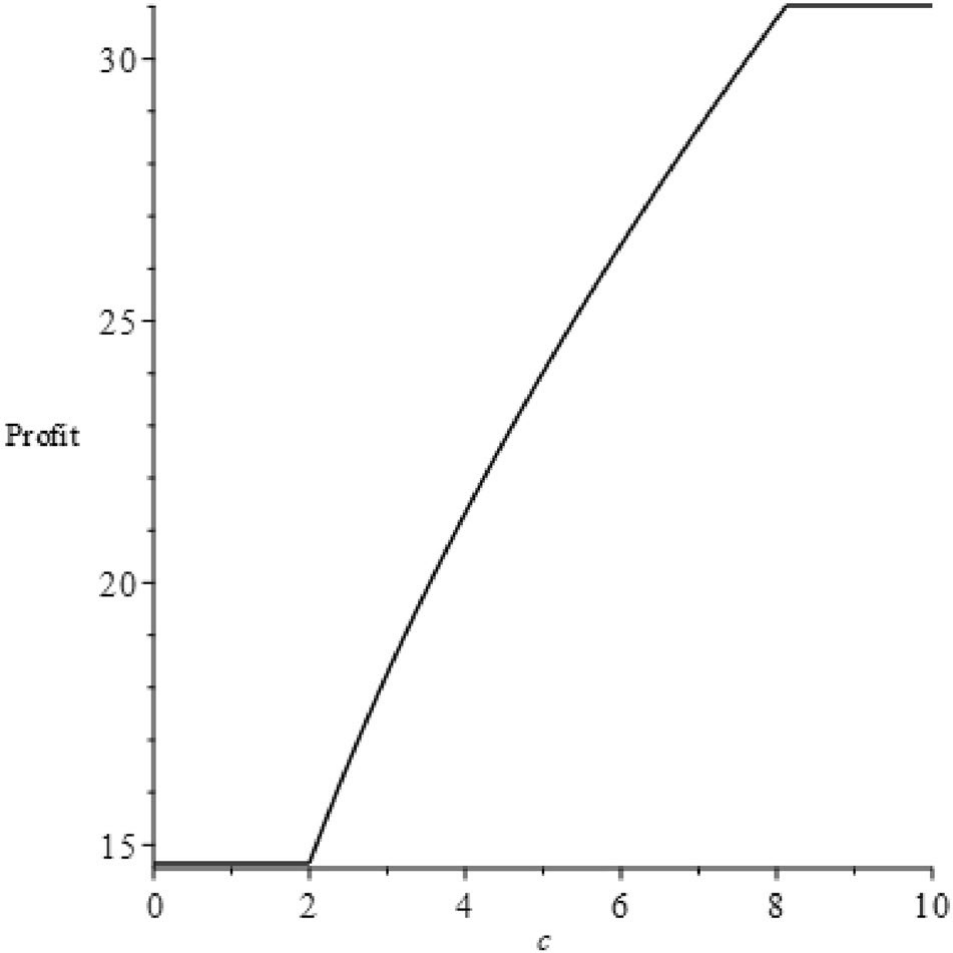
Profit changes according to $c$ for the first part of the proposition.

**Figure 6:**
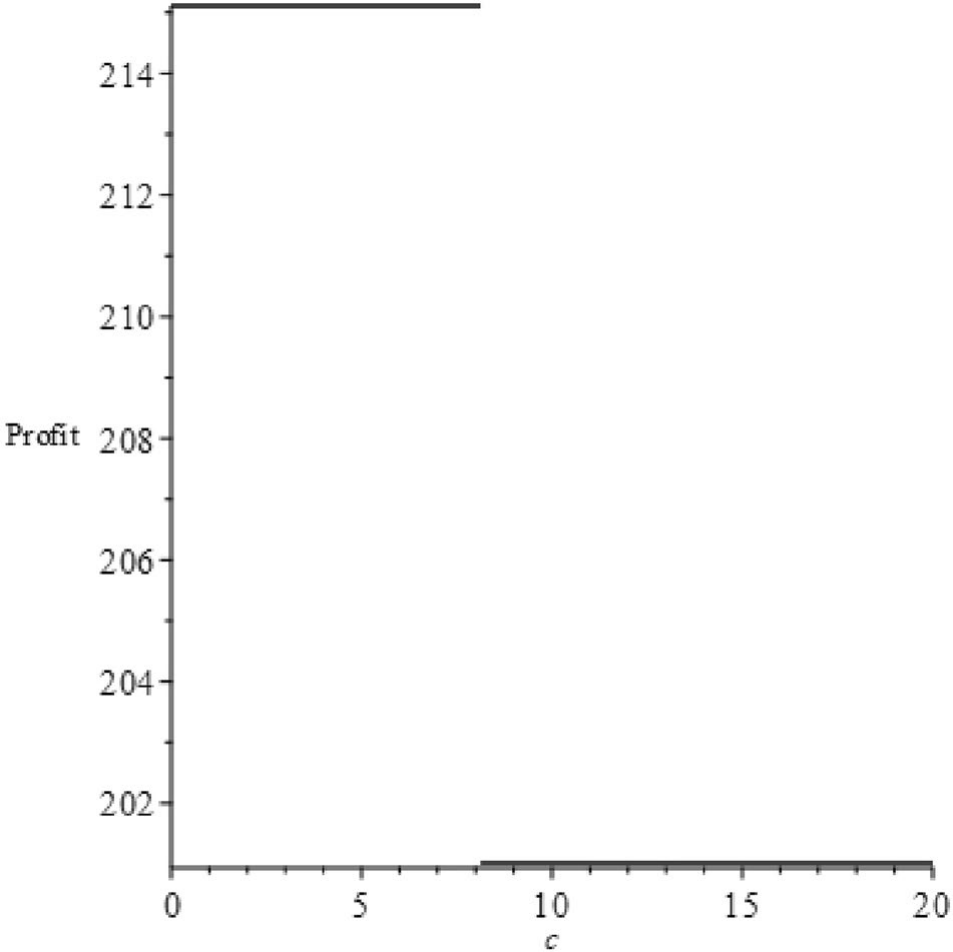
Profit changes according to $c$ for the second part of the proposition.

The proof is found in the Appendix. We now depict these trends vividly using figures. First, we let $v=60$, $b=65$ and $\alpha =300$, satisfying the condition in the proposition's first part. Next, we let $v=230$, $b=65$ and $\alpha =300$, adhering to the condition in the proposition's second part. Figures 5 and 6 illustrate the profit trends under these two conditions.

As seen from Fig. 5, when the condition $\frac{(4\alpha -b-\sqrt{2\alpha (4\alpha -b)})b}{2\alpha }\le v\le \frac{(4\alpha -b+\sqrt{2\alpha (4\alpha -b)})b}{2\alpha }$ is met, profits initially remain constant, then increase, and eventually stabilize. This trend is continuous. Conversely, Fig. 6 illustrates that profits remain constant initially, then decline to a low level and stabilize. As discussed earlier, there are two or three manufacturer decisions dictated by varying $c$ values. If the first part of the proposition is fulfilled, the manufacturer has three options. If the second part's condition is met, only two options exist. From Proposition 3, we learn that in most scenarios, profits don't depend on $c$. Profits can only increase if $\frac{((-4b+2v)\alpha +{b}^{2}{)}^{4}}{32{\alpha }^{2}{b}^{3}(-4\alpha +b{)}^{2}}<c\le \frac{b}{8}$. This is because, in this scenario, all consumers buy the product directly without investigation, keeping the demand unchanged. Yet, the product price increases due to $c$, as the decision is $$\left\{\begin{array}{l}{p}_{Sd}^{*}=\frac{2\sqrt{2bc}\alpha -2b\alpha +2v\alpha +{b}^{2}}{2\alpha }\\ {q}_{Sd}^{*}=\frac{b}{2\alpha }\end{array}\right.$$. However, according to Proposition 4, in most cases, the expected utility decreases with $c$, the only exception being at the point $c=\frac{((-4b+2v)\alpha +{b}^{2}{)}^{4}}{32{\alpha }^{2}{b}^{3}(-4\alpha +b{)}^{2}}$. Therefore, consumers favor lower investigation costs, potentially creating a contradiction between consumer and manufacturer preferences regarding the investigation cost.

### NGO-label scenario

The manufacturer's pricing decision always hinges on the NGO-label's value. After analyzing the results, the manufacturer's decisions are as follows:

#### Proposition 7

When $q\le 2-\frac{v}{b}$, the manufacturer decides on ${p}_{N}^{*}=\frac{v+bq}{2}$. The corresponding profit and utility are ${\pi }_{N}^{*}=\frac{(v+bq{)}^{2}}{4b}-\alpha {q}^{2}$ and ${U}_{N}^{*}=\frac{(v+bq{)}^{2}}{8b}$. When $q>2-\frac{v}{b}$, the manufacturer decides on ${p}_{N}^{*}=v+bq-b$. The corresponding profit and utility are ${\pi }_{N}^{*}=v+b(q-1)-\alpha {q}^{2}$ and ${U}_{N}^{*}=\frac{b}{2}$.

The proof is provided in the Appendix. From this proposition, we observe that the manufacturer has two choices based on different NGO-label values. However, regardless of the choice, the product price always increases with the NGO-label. From the above, we propose the following:

#### Proposition 8

Utility increases for $q$ within the range $\left[{0,2}-\frac{v}{b}\right]$ and remains constant for $q$ within $(2-\frac{v}{b},1]$.

Proof is in the Appendix. This proposition, however, suggests in the NGO-label scenario that if the label becomes too large (exceeding $2-\frac{v}{b}$), it ceases to impact the expected consumer utility. This is because, in such a case, ${p}_{N}^{*}=v+bq-b$ indicates that price also increases with $q$, rendering the expected utility constant. Thus, NGOs, although capable of setting high green standards to make products greener, cannot improve consumer utility. Overly stringent green standards can diminish the manufacturer's profit due to increased production costs, indicating neither consumers nor manufacturers prefer increasingly rigorous labels.

#### Proposition 9

If $v\le \frac{(4\alpha -b)}{2\alpha }$, the profit behaves as an increasing function for $q\in \left[0,\frac{v}{4\alpha -b}\right]$ and as a decreasing function for $q\in (\frac{v}{4\alpha -b},1]$. Alternatively, if this condition is not met, the profit exhibits an increasing trend for $q\in \left[0,\frac{b}{2\alpha }\right]$ and a decreasing trend for $q\in (\frac{b}{2\alpha },1]$.

**Figure 7:**
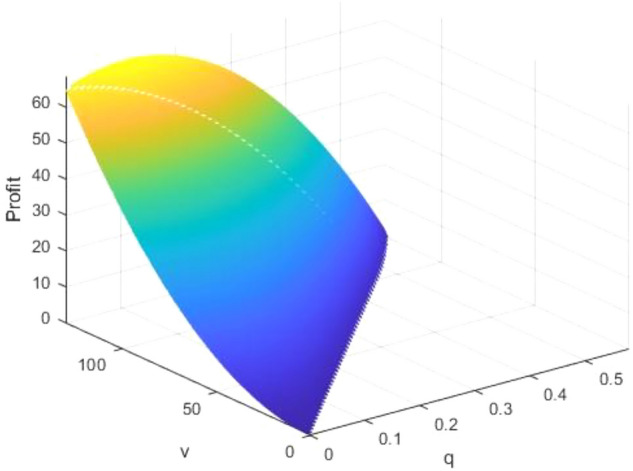
Profit changes according to $v$ and $q$.

The details of the proof are provided in the Appendix. To more clearly illustrate these trends, we consider a scenario where $b=65$ and $\alpha =300$. The variations in profit corresponding to changes in $v$ and $q$ are graphically represented in Fig. 7.

Proposition 9 and Fig. 7 collectively reveal that profit consistently exhibits a pattern of initial increase followed by a decrease in relation to $q$. During the intervals of increase, the manufacturer is inclined to accommodate the NGO's imposition of high label standards for the product. This is largely because an increase in q can potentially attract a larger consumer base. However, there exist certain intervals where the manufacturer may resist higher label standards, as excessive 'greenness' could entail substantial production costs. These conditions are shaped by variables $v$, $b$ and $\alpha$. Therefore, when an NGO sets the label standard, it should conduct a meticulous assessment of the green product market. This ensures a harmonious collaboration between the manufacturer and the NGO, promoting mutual benefits.

Proposition 10

Assuming $\frac{((-4b+2v)\alpha +{b}^{2}{)}^{4}}{32{\alpha }^{2}{b}^{3}(-4\alpha +b{)}^{2}}\le \frac{b}{8}$ and $q\le 2-\frac{v}{b}$, we can identify $c^{{\prime}}$,${c}^{{\prime\prime}}$,$c^{{\prime\prime\prime}}$ such that the expected utility in the self-label scenario exceeds the NGO-label scenario for $0\le c\le c^{{\prime}}$ and $c^{{\prime\prime}}\le c\le c^{{\prime\prime\prime}}$, where.

$$\left\{\begin{array}{l}c{^{\prime}}={\min}\left(\frac{((-4b+2v)\alpha +{b}^{2}{)}^{4}}{32{\alpha }^{2}{b}^{3}(-4\alpha +b{)}^{2}},\frac{\left(4\alpha q-bq-v\right)\left({b}^{2}q+\left(v-4\alpha q\right)b-8v\alpha \right)}{8{\left(b-4\alpha \right)}^{2}}\right)\\ c{^{\prime \prime}}=\frac{((-4b+2v)\alpha +{b}^{2}{)}^{4}}{32{\alpha }^{2}{b}^{3}(-4\alpha +b{)}^{2}} \\ c{^{\prime \prime \prime}}={\min}\left(\frac{b}{8},\frac{{\left(\left(q+2\right)b+v\right)}^{2}{\left(\left(q-2\right)b+v\right)}^{2}}{128{b}^{3}}\right)\end{array}\right.$$

Otherwise, the self-label scenario only yields a higher utility for $0\le c\le c^{{\prime}}$, where.

$${c}^{{{\prime}}}=\left\{\begin{array}{l}{\min}\left(\frac{b}{8},\frac{\left(4\alpha q-bq-v\right)\left({b}^{2}q+\left(v-4\alpha q\right)b-8v\alpha \right)}{8{\left(b-4\alpha \right)}^{2}}\right) when \frac{((-4b+2v)\alpha +{b}^{2}{)}^{4}}{32{\alpha }^{2}{b}^{3}(-4\alpha +b{)}^{2}}>\frac{b}{8}\mathrm{ and }q\le 2-\frac{v}{b}\\ {\min}\left(\frac{((-4b+2v)\alpha +{b}^{2}{)}^{4}}{32{\alpha }^{2}{b}^{3}(-4\alpha +b{)}^{2}},\frac{4{\alpha }^{2}{v}^{2}-16{\alpha }^{2}{b}^{2}+8\alpha {b}^{3}-{b}^{4}}{2{\left(-4\alpha +b\right)}^{2}b}\right) when \frac{((-4b+2v)\alpha +{b}^{2}{)}^{4}}{32{\alpha }^{2}{b}^{3}(-4\alpha +b{)}^{2}}\le \frac{b}{8}\mathrm{ and }q>2-\frac{v}{b}\\ {\min}\left(\frac{v}{b},\frac{4{\alpha }^{2}{v}^{2}-16{\alpha }^{2}{b}^{2}+8\alpha {b}^{3}-{b}^{4}}{2{\left(-4\alpha +b\right)}^{2}b}\right) when \frac{((-4b+2v)\alpha +{b}^{2}{)}^{4}}{32{\alpha }^{2}{b}^{3}(-4\alpha +b{)}^{2}}>\frac{b}{8}\mathrm{ and }q>2-\frac{v}{b}\end{array}\right.$$

The proof resides in the Appendix. For investigation costs satisfying $0\le c\le c^{{\prime}}$, self-label utility naturally surpasses NGO-label utility. Another interval for $c$, i.e., $c^{{\prime\prime}}\le c\le c^{{\prime\prime\prime}}$, allows the self-label scenario to outperform the NGO-label one. As per Proposition 4, manufacturers increase product greenness, ensuring direct purchases without consumer investigation, which boosts the self-label utility at $c=\frac{((-4b+2v)\alpha +{b}^{2}{)}^{4}}{32{\alpha }^{2}{b}^{3}(-4\alpha +b{)}^{2}}$

**Figure 8:**
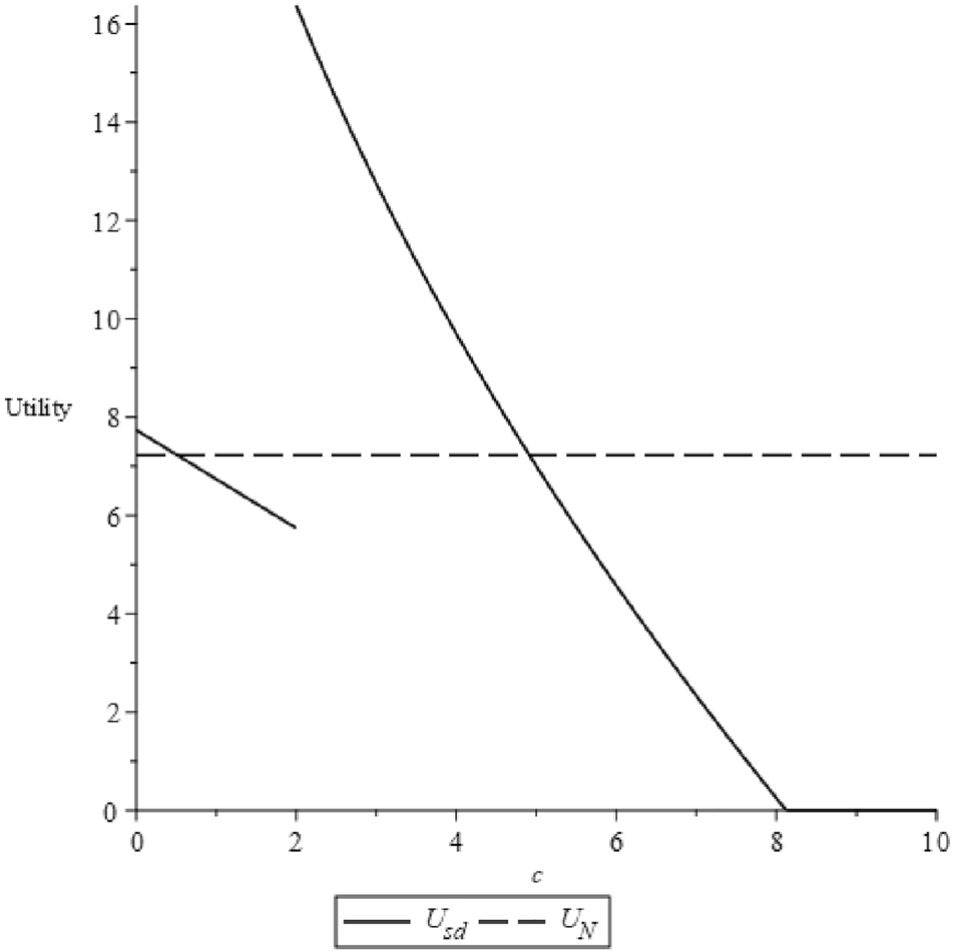
The trend of utility varying with $c$ under two eco-label scenarios.

To make the results more intuitive, we graphically depict the propositions. Based on the related settings in the previous section, let $q=0.02$, $v=60$, $b=65$ and $\alpha =300$, which satisfy $\frac{((-4b+2v)\alpha +{b}^{2}{)}^{4}}{32{\alpha }^{2}{b}^{3}(-4\alpha +b{)}^{2}}\le \frac{b}{8}$ and $q\le 2-\frac{v}{b}$. Accordingly, we can calculate that $c^{{\prime}}=0.51$, $c^{{\prime\prime}}=1.99$, $c^{{\prime\prime\prime}}=4.61$. Figure 8 displays the trend of consumer utility varying with the investigation cost under two ecological label scenarios.

Despite the investigation cost in the self-label scenario, this proposition suggests its potential to outpace the NGO-label scenario in terms of expected utility, deterring consumer acceptance of the NGO label. However, consumer behavior is steered by the manufacturer's decision. The profit comparison for both scenarios is presented below.

#### Proposition 11

For $c>\frac{((-4b+2v)\alpha +{b}^{2}{)}^{4}}{32{\alpha }^{2}{b}^{3}(-4\alpha +b{)}^{2}}$, there exists an $s$, expressed as $$\left\{\begin{array}{l}s=4b\alpha \sqrt{2bc}(b-4\alpha )+((2v-4b)\alpha +{b}^{2}{)}^{2}\text{ when }\frac{((-4b+2v)\alpha +{b}^{2}{)}^{4}}{32{\alpha }^{2}{b}^{3}(-4\alpha +b{)}^{2}}\le c\le \frac{b}{8} \\ s={b}^{4}-6\alpha {b}^{3}+4\alpha (v+2\alpha ){b}^{2}-16{\alpha }^{2}bv+4{\alpha }^{2}{v}^{2}\text{ when }c>\frac{b}{8}\end{array}\right.$$ When $s>0$, $q{\prime}$ and $q{\prime}{\prime}$ exist such that the NGO-label scenario only surpasses the self-label scenario in profit for $q{\prime}\le q\le q{\prime}{\prime}$, where $$\left\{\begin{array}{l}q{\prime}=\frac{\alpha bv-\sqrt{b\alpha s}}{\alpha b(4\alpha -b)} \, \\ q{\prime}{\prime}={\min}\left(\frac{\alpha bv-\sqrt{b\alpha s}}{\alpha b(4\alpha -b)},2-\frac{v}{b}\right)\end{array}\right.$$. Otherwise, the former scenario consistently falls short of the latter.

The proof is detailed in the Appendix. This finding suggests that an NGO cannot set its label standards excessively high or low. As per Proposition 9, profits in the NGO-label scenario initially rise and then fall with $q$. Hence, overly stringent or lax green standards yield lesser profits than the self-label scenario, leading to an NGO's failure in enticing manufacturers to adopt its green standards. Thus, an optimal $q$ range should be established to incentivize manufacturers to use the NGO-label for better profits. We observe that if a manufacturer's decision prompts consumer investigation in the self-label scenario, i.e., $c<\frac{((-4b+2v)\alpha +{b}^{2}{)}^{4}}{32{\alpha }^{2}{b}^{3}(-4\alpha +b{)}^{2}}$, then, according to Proposition 11, the manufacturer would reject the NGO-label. Referring to Proposition 6, we infer that only when $v<\frac{(4\alpha -b-\sqrt{2\alpha (4\alpha -b)})b}{2\alpha }$ or $v>\frac{(4\alpha -b+\sqrt{2\alpha (4\alpha -b)})b}{2\alpha }$ does the profit decrease at $c=\frac{b}{8}$. In other circumstances, neither the manufacturer nor NGO favors low investigation costs in the self-label scenario. Intriguingly, certain situations discourage manufacturers from advertising their own labels to minimize consumers' investigation costs.

**Figure 9:**
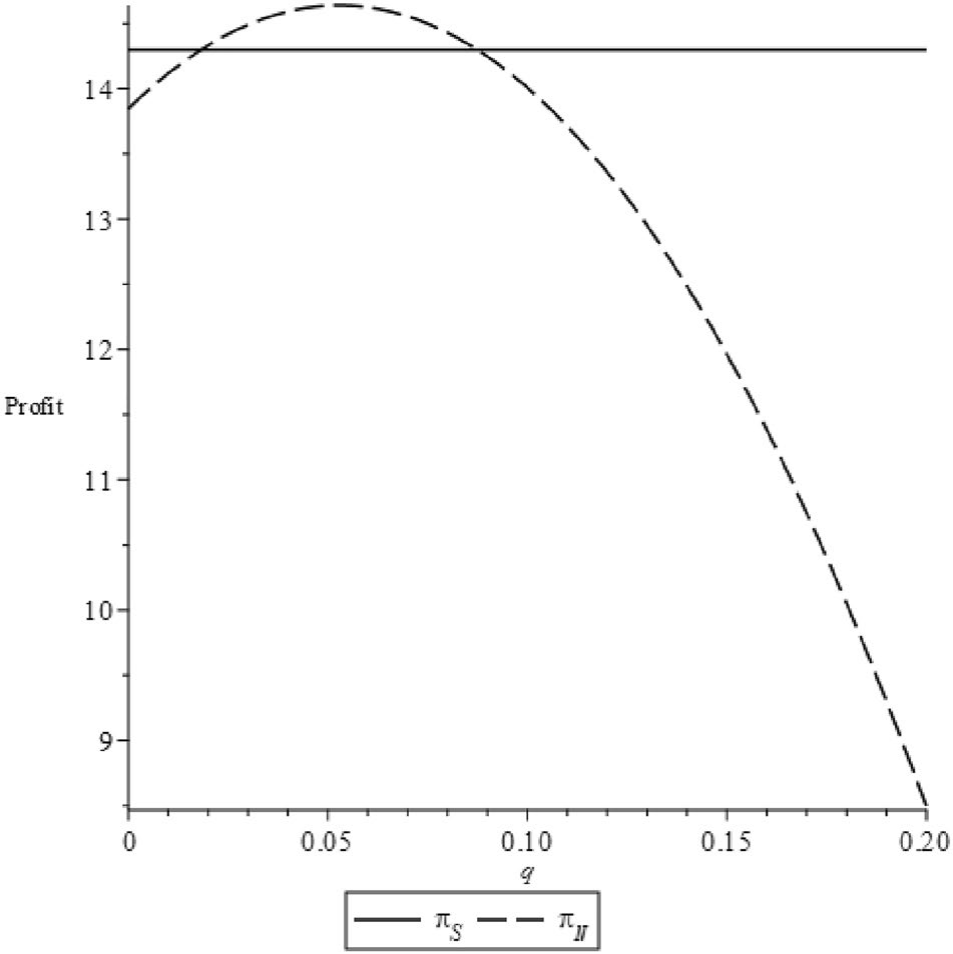
The trend of profit varying with $q$ under two eco-label scenarios.

We can leverage graphical representation to make the proposition clearer. Adopting the previous assumptions and setting $c=2$, $v=60$, $b=65$ and $\alpha =300$, we can calculate that ${q}{\prime}=0.018$ and $q{\prime}{\prime}=0.087$. Consequently, the profit trends varying with q under both the NGO-label and self-label scenarios are shown in Fig. 9.

## Conclusions and implications

This study explores decision-making within green supply chains under the backdrop of uncertain consumer environmental awareness and an eco-label policy. We investigate the effects of self-labeled and NGO-labeled products on consumer purchase decisions, deciphering consumer decision paths when faced with varied eco-labeled products. Furthermore, we contrast manufacturers' production and pricing decisions across both scenarios. Through analytical solutions for optimal pricing, product greenness, consumer utility, and manufacturers' profits, we deduce propositions for manufacturers and NGOs. These propositions elucidate insightful and practical management implications, directing manufacturers and NGOs in their eco-label determinations.

In the self-label scenario, we observe: (1) Consumer investigation behavior requires the cost of such behavior to be as low as possible. However, if the product's price itself is quite low, consumers would proceed to purchase directly without prior investigation of the product. Correspondingly, an example is Tesla electric cars. When their prices are reduced, sales quickly increase as consumers are attracted by its advanced performance and low price, not merely because it is an environmentally friendly electric car. (https://www.reuters.com/business/autos-transportation/tesla-cuts-prices-electric-vehicles-us-market-2023-01-13/) IKEA's LED light bulbs are often chosen by consumers due to their low cost. Consumers might first be attracted by its price, and then realize that they are actually choosing an environmentally friendly product. (https://www.ikea.com/us/en/this-is-ikea/about-us/a-sustainable-ikea-pubfa9dcf80) (2) Despite having a choice to investigate before buying, consumer behavior within the supply chain is ultimately swayed by the manufacturer's production and pricing choices. As investigation costs escalate, the manufacturer's decision nudges consumers away from investigating, leading to direct purchases. Khuzaimah et al.^60^, through empirical research on the Malaysian consumer market, confirmed the viewpoint that consumers might choose to purchase green products without fully understanding the environmental benefits of such products. In some specific valuation intervals, higher investigation costs may enhance consumer utility via increased product greenness and bolster manufacturers' profits through increased pricing. (3) Product greenness decreases at select investigation cost values if the manufacturer adjusts its decision. Typically, product greenness remains constant.

In the NGO-label scenario, we find: (1) As consumers do not need to investigate the product and its greenness solely depends on the NGO’s standards, buying behavior is solely influenced by the product price. Hence, when the price is sufficiently low, all consumers purchase the product; as the price increases, only those with a high perception of product greenness buy it, and at too high a price, no consumer purchases. (2) Consumer utility does not always elevate as the product gets greener. Excessively high greenness maintains consumer utility at a constant due to the corresponding higher price. For instance, many organic products, such as organic vegetables, fruits, and meats, usually have a much higher price than non-organic foods. Some consumers might feel that while organic food is healthier and more environmentally friendly, its high price and perceived lack of additional utility compared to non-organic food make it less appealing^61^. (3) While heightened greenness might stimulate consumer demand, it also incurs higher costs, hence the manufacturer's profit exhibits an initial increase followed by a decrease. (4) When investigation costs are minimal, both consumers and manufacturers reject the NGO-label due to diminished utility and profit. For instance, the environmental benefits of reusable shopping bags are very straightforward, as consumers can directly see a reduction in plastic bag use. Therefore, manufacturers might not pursue eco-label certification, as it adds to costs and could potentially affect the product's price^62^.

This study also yields several managerial insights.

In the self-label scenario, we note: (1) While consumers have the option to investigate a product, the manufacturer can influence this choice by determining the price and level of greenness. (2) When consumers' product valuation lies within a specific range, they don't always resist higher investigation costs. This is because the manufacturer can offer higher utility to consumers by persuading them to purchase without investigation. (3) If the investigation cost lies within a certain range, the manufacturer may prefer its increase to gain more profit. However, within this range, consumers would rather lower the investigation cost to maximize their utility, creating a contradiction.

In the NGO-label scenario, we observe: (1) Even though the NGO can set a high green standard to increase product greenness, it may not necessarily enhance consumer utility. Excessively high green standards can decrease the manufacturer's profit due to increased production costs. Hence, neither consumers nor manufacturers prefer increasingly stringent labels. This implies the NGO must conduct thorough market research for the green product to foster effective collaboration with the manufacturer. (2) When both the NGO's green standards and consumers' investigation costs are low, consumers may not necessarily favor the NGO-label over the manufacturer's self-label. (3) The NGO can't set its standard too high or low as it may fail to incentivize the manufacturer to use the NGO-label. (4) If market conditions and green technology meet certain criteria, neither the manufacturer nor NGO may prefer low investigation costs. Thus, manufacturers may not always seek to advertise their own labels to reduce consumer investigation costs.

Despite this paper's contribution to the eco-label supply chain decision support literature, future work can explore several interesting extensions. Firstly, treating the NGO's green standard as an exogenous variable for the manufacturer warrants detailed analysis of the game mechanics between the NGO and manufacturer. Secondly, to clarify our conclusions, we consider only one manufacturer and NGO. A competition scenario involving multiple manufacturers and NGOs regarding eco-labels would reveal additional managerial implications. Finally, expanding the supply chain's optimization objective to incorporate factors like social benefits into manufacturers' and NGOs' decision-making process is a focal point of our future research.

### Supplementary Information

Supplementary Information.

## Data Availability

All data generated or analysed during this study are included in this published article [and its supplementary information files].
