# Author Correction: Supply chain management with uncertainty in consumer perception of product greenness under an eco-label policy

**DOI:** 10.1038/s41598-023-42587-z

**Published:** 2023-09-18

**Authors:** Jingzhe Gao, Haixiao Wei

**Affiliations:** 1 School of Management and E‑Business, Zhejiang Gongshang University, Hangzhou, 310018 Zhejiang China; 2 Modern Business Research Center of Zhejiang Gongshang University, Key Research Institute of Humanities and Social Sciences for Universities, Ministry of Education of China, Hangzhou, China; 3 School of Management, Hangzhou Dianzi University, Hangzhou, 310018 Zhejiang China

Correction to: *Scientific Reports* 10.1038/s41598-023-40348-6, published online 21 August 2023

The Acknowledgements section in the original version of this Article was incomplete.

“This paper is supported by Zhejiang Provincial Philosophy and Social Sciences Planning Project and Hangzhou Philosophy and Social Sciences Planning Project [grant number Z23JC066].”

now reads:

“This paper is supported by Zhejiang Provincial Philosophy and Social Sciences Planning Project, National Natural Science Foundation of China [grant number 72304246] and Hangzhou Philosophy and Social Sciences Planning Project [grant number Z23JC066].”

The original Article has been corrected.

