# Supplementary material for: Supply chain management with uncertainty in consumer perception of product greenness under an eco-label policy
Source: Sci Rep. 2023 Aug 21;13:13581. doi: 10.1038/s41598-023-40348-6 (PMC10442351; doi:10.1038/s41598-023-40348-6)
Supplement: Supplementary file 1 — Supplementary Information. [file 41598_2023_40348_MOESM1_ESM.pdf]

Online Companion for "Supply chain management with uncertainty in consumer perception of product greenness under an eco-label policy"

Jingzhe Gao[a,b], HaixiaoWei[c,*]

[a] *School of Management and E-Business, Zhejiang Gongshang University, Hangzhou, Zhejiang 310018, China*

[b] *Modern Business Research Center of Zhejiang Gongshang University, Key Research Institute of Humanities and Social Sciences for Universities, Ministry of Education of China*

[c] *School of Management, Hangzhou Dianzi University, Hangzhou, Zhejiang 310018, China*

This is an online companion of "Supply chain management with uncertainty in consumer perception of product greenness under an eco-label policy". The companion consists of the proofs of the propositions.

Appendix

Proof of Proposition 1

If consumers investigate the product and then decide to buy it, the condition $U_{Sib} \geq U_{Sio}$ must be satisfied. This equals $x \in (-\infty, \frac{v-p}{b} + q]$. However, we have assumed that $x \in [0,1]$. Thus, there are 3 situations for the interval of x in which the above two intervals have different relative positions. Because U_{Si} has three situations, we need to divide U_{Sdb} into the three situations to compare them.

Situation 1

When $p > v + bq$, there is no intersection between $(-\infty, \frac{v-p}{b} + q]$ and $[0,1]$. No consumers buy the product after investigating. This is shown as follows:

$$\begin{cases} \text{buy:} & x \in \emptyset \\ \text{do not buy:} & x \in [0,1] \end{cases}$$

In this way, the expected utility for investigating and demand are $U_{Si} = \int_0^1 U_{Sio} dx = -c$ and $D_{Si} = 0$. We find that $U_{Si} = -c \leq U_{Sd} = 0$. Therefore, consumers cannot investigate the product. If consumers buy the product directly, the condition can be calculated as $p \leq v + bq - \frac{1}{2}b$. However, we know that this situation needs $p > v + bq$. Thus, situation 1 does not exist.

Situation 2

When $v + bq - b \leq p \leq v + bq$, it can be calculated as $0 \leq \frac{v-p}{b} + q \leq 1$. Therefore, we can obtain the intervals for x when the consumers buy or do not buy the product after

[*] Corresponding author.

E-mail addresses: gaojingzhe92@qq.com (J. Gao), smilewhx@163.com (H. Wei).

investigating.

$$\begin{cases} \text{buy:} \quad x \in \left[0, \frac{v-p}{b} + q\right] \\ \text{do not buy:} \ x \in \left[\frac{v-p}{b} + q, 1\right] \end{cases}$$

Thus, the expected utility and demand are shown as follows:

$$U_{Si} = \int_0^{\frac{v-p}{b}+q} U_{Sib} dx + \int_{\frac{v-p}{b}+q}^{1} U_{Sio} dx = \frac{b^2q^2 + (-2pq + 2qv - 2c)b + (-v+p)^2}{2b}$$

$$D_{Si} = \int_0^{\frac{v-p}{b}+q} 1dx = \frac{v-p}{b} + q$$

Then, according to the conditions for consumers to investigate and buy the product, we can calculate $p \in \left[v + bq - b + \sqrt{2bc}, v + bq - \sqrt{2bc}\right]$. It is obvious that this constraint requires $c \leq \frac{b}{8}$ to make the interval exist. If consumers buy the product without investigating, it can be calculated that $p \in \left[v + bq - b, min\left(v + bq - \frac{1}{2}b, v + bq - b + \sqrt{2bc}\right)\right]$. We find that when $c \leq \frac{b}{8}$, $p \in \left[v + bq - b, v + bq - b + \sqrt{2bc}\right]$; when $c > \frac{b}{8}$, $p \in \left[v + bq - b, v + bq - \frac{1}{2}b\right]$.

Thus, if consumers choose to investigate and buy the product, the problem can be expressed as:

$$Max\, \pi = \frac{q(p - \alpha q)b - p(p - v)}{b}$$

$$s.t. \begin{cases} p - bq \geq v - b + \sqrt{2bc} \\ p - bq \leq v - \sqrt{2bc} \\ p \geq 0 \\ 0 \leq q \leq 1 \end{cases}$$

This is a constrained optimization problem with no linear objective function. To check whether it is a convex problem, the determinant of the Hessian matrix can be calculated as follows.

$$H(p,q) = \begin{pmatrix} -\frac{2}{b} & 1 \\ 1 & -2\alpha \end{pmatrix}$$

Hence, $det[H(p,q)] = \frac{4\alpha}{b} - 1$. Because $\alpha > v + b$, we find that this is a convex problem. Using Karush-Kuhn–Tucker conditions, it can be calculated that the optimal solution is $\begin{cases} p^* = \frac{2\alpha v}{4\alpha - b} \\ q^* = \frac{v}{4\alpha - b} \end{cases}$. Therefore, the profit and utility are $\pi^* = \frac{\alpha v^2}{(4\alpha - b)b}$ and $U^* = \frac{2\alpha^2 v^2}{(b - 4\alpha)^2 b} - c$.

If consumers choose to buy the product without investigating, the problem can be

expressed as:

when $c \le \frac{b}{8}$,

$$Max\ \pi = p - \alpha q^2$$

$$s.t. \begin{cases} p - bq \ge v - b \\ p - bq \le v - b + \sqrt{2bc} \\ p \ge 0 \\ 0 \le q \le 1 \end{cases}$$

when $c > \frac{b}{8}$,

$$Max\ \pi = p - \alpha q^2$$

$$s.t. \begin{cases} p - bq \ge v - b \\ p - bq \le v - \frac{1}{2}b \\ p \ge 0 \\ 0 \le q \le 1 \end{cases}$$

It is obvious that π is always a monotonic function. Therefore, the optimal value can only be achieved on the boundary of the feasible region. Using the Karush-Kuhn–Tucker conditions and $\alpha > v + b$, we can calculate that when $c \le \frac{b}{8}$, the optimal solution is $\begin{cases} p^* = \frac{2\sqrt{2bc}\alpha - 2b\alpha + 2v\alpha + b^2}{2\alpha} \\ q^* = \frac{b}{2\alpha} \end{cases}$. The corresponding profit and utility are $\pi^* = \frac{4\sqrt{2bc}\alpha + 4(v-b)\alpha + b^2}{4\alpha}$ and $U^* = \frac{b}{2} - \sqrt{2bc}$. When $c > \frac{b}{8}$, the optimal solution is $\begin{cases} p^* = -\frac{b\alpha - 2v\alpha - b^2}{2\alpha} \\ q^* = \frac{b}{2\alpha} \end{cases}$. The corresponding profit and utility are $\pi^* = \frac{(4v-2b)\alpha + b^2}{4\alpha}$ and $U^* = 0$.

Situation 3

When $p \le v + bq - b$, it can be calculated as $1 \le \frac{v-p}{b} + q$. Therefore, the intervals of x when the consumers buy or do not buy the product after investigating can be calculated as:

$$\begin{cases} \text{buy: } x \in [0,1] \\ \text{do not buy: } x \in \emptyset \end{cases}$$

The expected utility for investigation and demand are $U_{Si} = \int_0^1 U_{Sib}\, dx = v + bq - p - c - \frac{1}{2}b$ and $D_{Si} = \int_0^1 1\, dx = 1$. Because $U_{Si} = v + bq - p - c - \frac{1}{2}b < U_{Sdb} = v + bq - p - \frac{1}{2}b$, consumers cannot investigate the product in this situation. If consumers buy the product directly, it can be calculated that $p \le v + bq - b$ needs to be satisfied.

Because in this situation, consumers can only buy the product directly without investigating, the problem can be expressed as:

$$Max\ \pi = p - \alpha q^2$$

$$s.t.\begin{cases} p - bq \leq v - b \\ p \geq 0 \\ 0 \leq q \leq 1 \end{cases}$$

π is always a monotonic function, so the optimal value can only be achieved on the boundary of the feasible region. Using the Karush-Kuhn–Tucker conditions and $\alpha > v + b$, we can calculate that $\begin{cases} p^* = -\frac{2b\alpha-2v\alpha-b^2}{2\alpha} \\ q^* = \frac{b}{2\alpha} \end{cases}$. The corresponding profit and utility are $\pi^* = \frac{(4v-4b)\alpha+b^2}{4\alpha}$ and $U^* = \frac{b}{2}$.

Thus, the proposition is proved.

Proof of Proposition 2

If consumers decide to buy the product, $U_{Nb} \geq U_{No}$ must be satisfied. This equals $x \leq \frac{v-p}{b} + q$. Therefore, 3 situations also exist, as in the self-label scenario, for the interval of x. The objective function can be shown as $Max\ \pi = pD - \alpha q^2$, and the manufacturer only needs to determine the value of p.

Situation 1

When $p > v + bq$, because there is no intersection between $(-\infty, \frac{v-p}{b} + q]$ and $[0,1]$, all consumers are unwilling to buy the product, which is shown as follows:

$$\begin{cases} \text{buy:} \quad x \in \emptyset \\ \text{do not buy:}\ x \in [0,1] \end{cases}$$

In this way, the expected utility and demand are $U_N = \int_0^1 U_{No}\, dx = 0$ and $D_N = 0$. Therefore, this situation does not exist.

Situation 2

When $v + bq - b \leq p \leq v + bq$, the intervals for x when the consumers buy or do not buy the product are shown as follows:

$$\begin{cases} \text{buy:} \quad x \in \left[0, \frac{v-p}{b} + q\right] \\ \text{do not buy:}\ x \in \left[\frac{v-p}{b} + q, 1\right] \end{cases}$$

Thus, the expected utility and demand are

$$U_N = \int_0^{\frac{v-p}{b}+q} U_{Nb} dx + \int_{\frac{v-p}{b}+q}^{1} U_{No} dx = \frac{(qb - p + v)^2}{2b}$$

$$D_N = \int_0^{\frac{v-p}{b}+q} 1dx = \frac{v-p}{b} + q$$

Thus, the problem can be shown as:

$$Max\ \pi = \frac{q(p - \alpha q)b - p(p - v)}{b}$$

$$s.t.\begin{cases} v+bq-b \le p \le v+bq \\ p \ge 0 \end{cases}$$

This is a constrained optimization problem with no linear objective function. We calculate that $\frac{\partial^2 \pi}{\partial p^2} = -\frac{2}{b}$. Therefore, this is a convex problem. Using the first-order condition, we obtain $p^* = \frac{v+bq}{2}$. The corresponding profit and utility are $\pi^* = \frac{(v+bq)^2}{4b} - \alpha q^2$ and $U^* = \frac{(v+bq)^2}{8b}$. However, only when $0 \le q \le 2-\frac{v}{b}$ can this solution satisfy the constraints. Otherwise, we need to consider $\frac{v+bq}{2} < v+bq-b$. Now, in the interval of p, profit is obviously a decreasing function. Therefore, when $q > 2-\frac{v}{b}$, the optimal solution is $p^* = v+bq-b$. The profit and utility are $\pi^* = v+b(q-1)-\alpha q^2$ and $U^* = \frac{b}{2}$.

Situation 3

When $p \le v+bq-b$, the intervals for x when the consumers buy or do not buy the product can be calculated as:

$$\begin{cases} \text{buy: } x \in [0,1] \\ \text{do not buy: } x \in \emptyset \end{cases}$$

Thus, consumers would always buy it. The expected utility and demand are $U_N = \int_0^1 U_{Nb}\, dx = v+bq-p-\frac{1}{2}b$ and $D_N = \int_0^1 1\, dx = 1$. The problem can be expressed as:

$$Max\, \pi = p - \alpha q^2$$
$$s.t. 0 \le p \le v+bq-b$$

This is obviously an increasing function of p. Therefore, the optimal solution must be $p^* = v+bq-b$. The corresponding profit and utility are $\pi^* = v+b(q-1)-\alpha q^2$ and $U^* = \frac{b}{2}$. This requires $v+bq-b \ge 0$, which equals $q \ge 1-\frac{v}{b}$. Otherwise, the constraints for p would never be satisfied, and this situation does not exist.

Thus, the proposition is proved.

Proof of Proposition 3

According to the optimal solutions in this scenario, we find that when consumers choose not to investigate the product, the profit in situation 2 is always larger than that in situation 3. Thus, the solutions in situation 3 cannot be optimal. The manufacturer only needs to compare the decisions in situation 2. When $c \le \frac{b}{8}$, there exist two solutions such as $\begin{cases} p_{Si}^* = \frac{2\alpha v}{4\alpha - b} \\ q_{Si}^* = \frac{v}{4\alpha - b} \end{cases}$ and $\begin{cases} p_{Sd}^* = \frac{2\sqrt{2bc}\alpha - 2b\alpha + 2v\alpha + b^2}{2\alpha} \\ q_{Sd}^* = \frac{b}{2\alpha} \end{cases}$. The corresponding profits are $\pi_{Si}^* = \frac{\alpha v^2}{(4\alpha - b)b}$ and $\pi_{Sd}^* = \frac{4\sqrt{2bc}\alpha + 4(v-b)\alpha + b^2}{4\alpha}$. If $c \le \frac{((-4b+2v)\alpha + b^2)^4}{32\alpha^2 b^3(-4\alpha+b)^2}$, the former is the largest. Otherwise, the latter is the largest. Then, the utilities are $U_{Si}^* = \frac{2\alpha^2 v^2}{(b-4\alpha)^2 b} - c$ and $U_{Sd}^* = \frac{b}{2} - \sqrt{2bc}$, respectively. When

$c > \frac{b}{8}$, there exists only one solution, such as $\begin{cases} p_{Sd}^* = -\frac{b\alpha - 2v\alpha - b^2}{2\alpha} \\ q_{Sd}^* = \frac{b}{2\alpha} \end{cases}$. The corresponding profit and utility are $\pi_{Sd}^* = \frac{(4v-2b)\alpha + b^2}{4\alpha}$ and $U_{Sd}^* = 0$. Thus, the proposition is proved.

Proof of Proposition 4

According to Proposition 3, if $\frac{((-4b+2v)\alpha+b^2)^4}{32\alpha^2 b^3(-4\alpha+b)^2} \leq \frac{b}{8}$ which equals $\frac{(4\alpha-b-\sqrt{2\alpha(4\alpha-b)})b}{2\alpha} \leq v \leq \frac{(4\alpha-b+\sqrt{2\alpha(4\alpha-b)})b}{2\alpha}$, there exist three utility functions including $U_{Si}^* = \frac{2\alpha^2 v^2}{(b-4\alpha)^2 b} - c$ in $c \in \left[0, \frac{((-4b+2v)\alpha+b^2)^4}{32\alpha^2 b^3(-4\alpha+b)^2}\right]$, $U_{Sd}^* = \frac{b}{2} - \sqrt{2bc}$ in $c \in (\frac{((-4b+2v)\alpha+b^2)^4}{32\alpha^2 b^3(-4\alpha+b)^2}, \frac{b}{8}]$, and $U_{Sd}^* = 0$ in $c \in \left(\frac{b}{8}, +\infty\right)$. The first and second are both decreasing functions of c. Therefore, we need to investigate the two points $c = \frac{((-4b+2v)\alpha+b^2)^4}{32\alpha^2 b^3(-4\alpha+b)^2}$ and $c = \frac{b}{8}$. It can be calculated that if $0 \leq v < \frac{b(4\alpha-b)}{2\alpha}$, when $\frac{(4\alpha b-2\alpha v-b^2)^2}{2(4\alpha-b)^2 b} \leq c \leq \frac{b}{8}$, $U_{Si}^* = \frac{2\alpha^2 v^2}{(b-4\alpha)^2 b} - c \geq U_{Sd}^* = \frac{b}{2} - \sqrt{2bc}$; when $c < \frac{(4\alpha b-2\alpha v-b^2)^2}{2(4\alpha-b)^2 b}$, $U_{Si}^* = \frac{2\alpha^2 v^2}{(b-4\alpha)^2 b} - c < U_{Sd}^* = \frac{b}{2} - \sqrt{2bc}$. If $v \geq \frac{b(4\alpha-b)}{2\alpha}$, then $U_{Si}^* > U_{Sd}^*$. Thus, we need to compare $\frac{(4\alpha b-2\alpha v-b^2)^2}{2(4\alpha-b)^2 b}$ and $\frac{((-4b+2v)\alpha+b^2)^4}{32\alpha^2 b^3(-4\alpha+b)^2}$ with $\frac{b}{8}$. However, when $0 \leq v < \frac{b(4\alpha-b)}{2\alpha}$ and $\alpha > v + b$, it can be calculated that $\frac{(4\alpha b-2\alpha v-b^2)^2}{2(4\alpha-b)^2 b} > \frac{((-4b+2v)\alpha+b^2)^4}{32\alpha^2 b^3(-4\alpha+b)^2}$. This means the utility would increase at point $c = \frac{((-4b+2v)\alpha+b^2)^4}{32\alpha^2 b^3(-4\alpha+b)^2}$. Otherwise, the utility would decrease at this point. In addition, if $\frac{((-4b+2v)\alpha+b^2)^4}{32\alpha^2 b^3(-4\alpha+b)^2} \leq \frac{b}{8}$, utility $U_{Sd}^* = \frac{b}{2} - \sqrt{2bc}$ equals zero when $c = \frac{b}{8}$.

If $\frac{((-4b+2v)\alpha+b^2)^4}{32\alpha^2 b^3(-4\alpha+b)^2} > \frac{b}{8}$, which equals $v < \frac{(4\alpha-b-\sqrt{2\alpha(4\alpha-b)})b}{2\alpha}$ or $v > \frac{(4\alpha-b+\sqrt{2\alpha(4\alpha-b)})b}{2\alpha}$, utility $U_{Sd}^* = \frac{b}{2} - \sqrt{2bc}$ does not exist, and $U_{Si}^* = \frac{2\alpha^2 v^2}{(b-4\alpha)^2 b} - c$ would decrease to $U_{Sd}^* = 0$ directly when $c = \frac{b}{8}$.

In this way, the trend of the utility in this proposition is proved.

Proof of Proposition 5

Suppose that $\frac{((-4b+2v)\alpha+b^2)^4}{32\alpha^2 b^3(-4\alpha+b)^2} \leq \frac{b}{8}$, which equals $\frac{(4\alpha-b-\sqrt{2\alpha(4\alpha-b)})b}{2\alpha} \leq v \leq \frac{(4\alpha-b+\sqrt{2\alpha(4\alpha-b)})b}{2\alpha}$; the optimal greenness would change from $q_{Si}^* = \frac{v}{4\alpha-b}$ to $q_{Sd}^* = \frac{b}{2\alpha}$ at $c = \frac{((-4b+2v)\alpha+b^2)^4}{32\alpha^2 b^3(-4\alpha+b)^2}$. Otherwise, it would change from the former to the latter at $c = \frac{b}{8}$. Now,

we need to compare $q_{Si}^* = \frac{v}{4\alpha - b}$ with $q_{Sd}^* = \frac{b}{2\alpha}$. If $v \le \frac{b(4\alpha-b)}{2\alpha}$, then $q_{Si}^* \le q_{Sd}^*$. Otherwise, $q_{Si}^* > q_{Sd}^*$. Thus, the proposition is proved.

Proof of Proposition 6

According to Proposition 3, if $\frac{((-4b+2v)\alpha+b^2)^4}{32\alpha^2 b^3(-4\alpha+b)^2} \le \frac{b}{8}$, which equals $\frac{(4\alpha-b-\sqrt{2\alpha(4\alpha-b)})b}{2\alpha} \le v \le \frac{(4\alpha-b+\sqrt{2\alpha(4\alpha-b)})b}{2\alpha}$, there exist three profit functions, including $\pi_{Si}^* = \frac{\alpha v^2}{(4\alpha-b)b}$ in $c \in \left[0, \frac{((-4b+2v)\alpha+b^2)^4}{32\alpha^2 b^3(-4\alpha+b)^2}\right]$, $\pi_{Sd}^* = \frac{4\sqrt{2bc}\alpha+4(v-b)\alpha+b^2}{4\alpha}$ in $c \in (\frac{((-4b+2v)\alpha+b^2)^4}{32\alpha^2 b^3(-4\alpha+b)^2}, \frac{b}{8}]$, and $\pi_{Sd}^* = \frac{(4v-2b)\alpha+b^2}{4\alpha}$ in $c \in \left(\frac{b}{8}, +\infty\right)$. The first and third cannot be influenced by c. The second is an increasing function of c. It can be calculated that when $c = \frac{((-4b+2v)\alpha+b^2)^4}{32\alpha^2 b^3(-4\alpha+b)^2}$, $\pi_{Si}^* = \frac{\alpha v^2}{(4\alpha-b)b} = \pi_{Sd}^* = \frac{4\sqrt{2bc}\alpha+4(v-b)\alpha+b^2}{4\alpha}$; when $c = \frac{b}{8}$, $\pi_{Sd}^* = \frac{4\sqrt{2bc}\alpha+4(v-b)\alpha+b^2}{4\alpha} = \pi_{Sd}^* = \frac{(4v-2b)\alpha+b^2}{4\alpha}$. Therefore, the whole trend is continuous.

If $\frac{((-4b+2v)\alpha+b^2)^4}{32\alpha^2 b^3(-4\alpha+b)^2} > \frac{b}{8}$, there only exist two profit functions, such as $\pi_{Si}^* = \frac{\alpha v^2}{(4\alpha-b)b}$ in $c \in \left[0, \frac{b}{8}\right]$ and $\pi_{Sd}^* = \frac{(4v-2b)\alpha+b^2}{4\alpha}$ in $c \in \left(\frac{b}{8}, +\infty\right)$. Therefore, we need to compare these two profit functions. If $\frac{(4\alpha-b-\sqrt{2\alpha(4\alpha-b)})b}{2\alpha} \le v \le \frac{(4\alpha-b+\sqrt{2\alpha(4\alpha-b)})b}{2\alpha}$, then $\pi_{Si}^* = \frac{\alpha v^2}{(4\alpha-b)b} \le \pi_{Sd}^* = \frac{(4v-2b)\alpha+b^2}{4\alpha}$. However, this is impossible because $\frac{((-4b+2v)\alpha+b^2)^4}{32\alpha^2 b^3(-4\alpha+b)^2} > \frac{b}{8}$ equals $v < \frac{(4\alpha-b-\sqrt{2\alpha(4\alpha-b)})b}{2\alpha}$ or $v > \frac{(4\alpha-b+\sqrt{2\alpha(4\alpha-b)})b}{2\alpha}$. Therefore, the former is larger than the latter.

Thus, the proposition is proved.

Proof of Proposition 7

According to the solutions in this scenario, we find that when $q \le 1 - \frac{v}{b}$, the optimal profit is $\pi_N^* = \frac{(v+bq)^2}{4b} - \alpha q^2$. When $1 - \frac{v}{b} < q < 2 - \frac{v}{b}$, the manufacturer needs to compare $\pi_N^* = \frac{(v+bq)^2}{4b} - \alpha q^2$ with $\pi_N^* = v + b(q-1) - \alpha q^2$. It can be calculated that the former is larger than the latter. When $q > 2 - \frac{v}{b}$, the optimal profit is $\pi_N^* = v + b(q-1) - \alpha q^2$. Thus, the proposition is proved.

Proof of Proposition 8

According to Proposition 7, the utility would be $U_N^* = \frac{(v+bq)^2}{8b}$ in $q \in \left[0, 2 - \frac{v}{b}\right]$ and $U_N^* = \frac{b}{2}$ in $q \in \left(2 - \frac{v}{b}, +\infty\right)$. It is obvious that the former is an increasing function and the latter is a constant. When $q = 2 - \frac{v}{b}$, it can be calculated that the former equals the latter. Thus,

the proposition is proved.

Proof of Proposition 9

According to Proposition 7, if $q \le 2-\frac{v}{b}$, the profit is $\pi_N^* = \frac{(v+bq)^2}{4b} - \alpha q^2$. Because $v > b+\alpha$, it can be calculated that the profit is an increasing function in $q \in \left[0, \frac{v}{4\alpha-b}\right]$ and a decreasing function in $q \in \left(\frac{v}{4\alpha-b}, +\infty\right)$. If $q > 2-\frac{v}{b}$, the profit is $\pi_N^* = v + b(q-1) - \alpha q^2$. It can be calculated that the profit is an increasing function in $q \in \left[0, \frac{b}{2\alpha}\right]$ and a decreasing function in $q \in \left(\frac{b}{2\alpha}, +\infty\right)$. Thus, we need to compare $\frac{v}{4\alpha-b}$, $\frac{b}{2\alpha}$ with $2-\frac{v}{b}$. It can be calculated that $v \le \frac{(4\alpha-b)b}{2\alpha}$ leads to $\frac{v}{4\alpha-b} \le 2-\frac{v}{b}$ and $\frac{b}{2\alpha} \le 2-\frac{v}{b}$. Otherwise, we can obtain $\frac{v}{4\alpha-b} > 2-\frac{v}{b}$ and $\frac{b}{2\alpha} > 2-\frac{v}{b}$. Thus, the proposition is proved.

Proof of Proposition 10

If $\frac{((-4b+2v)\alpha+b^2)^4}{32\alpha^2 b^3(-4\alpha+b)^2} \le \frac{b}{8}$ and $q \le 2-\frac{v}{b}$, the expected utility in the NGO-label scenario is $U_N^* = \frac{(v+bq)^2}{8b}$. The utility in the self-label scenario is $U_{Si}^* = \frac{2\alpha^2 v^2}{(b-4\alpha)^2 b} - c$ in $c \in \left[0, \frac{((-4b+2v)\alpha+b^2)^4}{32\alpha^2 b^3(-4\alpha+b)^2}\right]$, $U_{Sd}^* = \frac{b}{2} - \sqrt{2bc}$ in $c \in (\frac{((-4b+2v)\alpha+b^2)^4}{32\alpha^2 b^3(-4\alpha+b)^2}, \frac{b}{8}]$ and $U_{Sd}^* = 0$ in $c \in \left(\frac{b}{8}, +\infty\right)$. First, we need to compare $U_N^* = \frac{(v+bq)^2}{8b}$ with $U_{Si}^* = \frac{2\alpha^2 v^2}{(b-4\alpha)^2 b} - c$. It can be calculated that $c' = min\left(\frac{((-4b+2v)\alpha+b^2)^4}{32\alpha^2 b^3(-4\alpha+b)^2}, \frac{(4\alpha q-bq-v)(b^2 q+(v-4\alpha q)b-8v\alpha)}{8(b-4\alpha)^2}\right)$. Second, we need to compare $U_N^* = \frac{(v+bq)^2}{8b}$ with $U_{Sd}^* = \frac{b}{2} - \sqrt{2bc}$. It can be calculated that $c'' = \frac{((-4b+2v)\alpha+b^2)^4}{32\alpha^2 b^3(-4\alpha+b)^2}$ and $c''' = min\left(\frac{b}{8}, \frac{\left((q+2)b+v\right)^2\left((q-2)b+v\right)^2}{128b^3}\right)$. In addition, it is obvious that $U_N^* = \frac{(v+bq)^2}{8b} \ge U_{Sd}^* = 0$.

If $\frac{((-4b+2v)\alpha+b^2)^4}{32\alpha^2 b^3(-4\alpha+b)^2} > \frac{b}{8}$ and $q \le 2-\frac{v}{b}$, the expected utility in the NGO-label scenario is $U_N^* = \frac{(v+bq)^2}{8b}$. The utility in the self-label scenario is $U_{Si}^* = \frac{2\alpha^2 v^2}{(b-4\alpha)^2 b} - c$ in $c \in \left[0, \frac{b}{8}\right]$ and $U_{Sd}^* = 0$ in $c \in \left(\frac{b}{8}, +\infty\right)$. Therefore, we only need to compare $U_N^* = \frac{(v+bq)^2}{8b}$ with $U_{Si}^* = \frac{2\alpha^2 v^2}{(b-4\alpha)^2 b} - c$. It can be calculated that $c' = min\left(\frac{b}{8}, \frac{(4\alpha q-bq-v)(b^2 q+(v-4\alpha q)b-8v\alpha)}{8(b-4\alpha)^2}\right)$.

If $\frac{((-4b+2v)\alpha+b^2)^4}{32\alpha^2 b^3(-4\alpha+b)^2} \le \frac{b}{8}$ and $q > 2-\frac{v}{b}$, the expected utility in the NGO-label scenario is $U_N^* = \frac{b}{2}$. It is obvious that $U_N^* = \frac{b}{2} > U_{Sd}^* = \frac{b}{2} - \sqrt{2bc}$ and $U_N^* = \frac{b}{2} > U_{Sd}^* = 0$. Therefore, we

only need to compare it with $U_{Si}^* = \frac{2\alpha^2 v^2}{(b-4\alpha)^2 b} - c$. It can be calculated that $c' = min\left(\frac{((-4b+2v)\alpha+b^2)^4}{32\alpha^2 b^3(-4\alpha+b)^2}, \frac{4\alpha^2v^2-16\alpha^2b^2+8\alpha b^3-b^4}{2(-4\alpha+b)^2 b}\right)$.

If $\frac{((-4b+2v)\alpha+b^2)^4}{32\alpha^2 b^3(-4\alpha+b)^2} > \frac{b}{8}$ and $q > 2 - \frac{v}{b}$, we also only need to compare $U_N^* = \frac{b}{2}$ with $U_{Si}^* = \frac{2\alpha^2 v^2}{(b-4\alpha)^2 b} - c$. It can be calculated that $c' = min\left(\frac{v}{b}, \frac{4\alpha^2v^2-16\alpha^2b^2+8\alpha b^3-b^4}{2(-4\alpha+b)^2 b}\right)$.

Thus, the proposition is proved.

Proof of Proposition 11

If $q \le 2 - \frac{v}{b}$, the profit in the NGO-label scenario is $\pi_N^* = \frac{(v+bq)^2}{4b} - \alpha q^2$. According to Proposition 3, the profit in the self-label scenario is $\pi_{Si}^* = \frac{\alpha v^2}{(4\alpha-b)b}$ in $c \in \left[0, \frac{((-4b+2v)\alpha+b^2)^4}{32\alpha^2b^3(-4\alpha+b)^2}\right]$, $\pi_{Sd}^* = \frac{4\sqrt{2bc}\alpha+4(v-b)\alpha+b^2}{4\alpha}$ in $c \in \left(\frac{((-4b+2v)\alpha+b^2)^4}{32\alpha^2b^3(-4\alpha+b)^2}, \frac{b}{8}\right]$, and $\pi_{Sd}^* = \frac{(4v-2b)\alpha+b^2}{4\alpha}$ in $c \in \left(\frac{b}{8}, +\infty\right)$. It can be calculated that $\pi_N^* = \frac{(v+bq)^2}{4b} - \alpha q^2 \le \pi_{Si}^* = \frac{\alpha v^2}{(4\alpha-b)b}$. Therefore, we only need to compare the remaining two profits with π_N^*. For $\pi_{Sd}^* = \frac{4\sqrt{2bc}\alpha+4(v-b)\alpha+b^2}{4\alpha}$ and $\pi_N^* = \frac{(v+bq)^2}{4b} - \alpha q^2$, it can be calculated that $s = 4b\alpha\sqrt{2bc}(b-4\alpha) + ((2v-4b)\alpha+b^2)^2$ and $\begin{cases} q' = \frac{\alpha bv-\sqrt{b\alpha s}}{\alpha b(4\alpha-b)} \\ q'' = min\left(\frac{\alpha bv-\sqrt{b\alpha s}}{\alpha b(4\alpha-b)}, 2-\frac{v}{b}\right) \end{cases}$. For $\pi_{Sd}^* = \frac{(4v-2b)\alpha+b^2}{4\alpha}$ and $\pi_N^* = \frac{(v+bq)^2}{4b} - \alpha q^2$, it can be calculated that $s = b^4 - 6\alpha b^3 + 4\alpha(v+2\alpha)b^2 - 16\alpha^2bv + 4\alpha^2v^2$ and $\begin{cases} q' = \frac{\alpha bv-\sqrt{b\alpha s}}{\alpha b(4\alpha-b)} \\ q'' = min\left(\frac{\alpha bv-\sqrt{b\alpha s}}{\alpha b(4\alpha-b)}, 2-\frac{v}{b}\right) \end{cases}$.

If $q > 2 - \frac{v}{b}$, the profit in the NGO-label scenario is $\pi_N^* = v + b(q-1) - \alpha q^2$. It can be calculated that this profit is always smaller than the three profits in the self-label scenario regardless of the value of the investigation cost. Thus, the proposition is proved.
